# Acetylation of ELF5 suppresses breast cancer progression by promoting its degradation and targeting *CCND1*

**DOI:** 10.1038/s41698-021-00158-3

**Published:** 2021-03-19

**Authors:** Xiahui Li, Shujing Li, Bowen Li, Yanan Li, Sattout Aman, Kangkai Xia, Yuxi Yang, Bashir Ahmad, Huijian Wu

**Affiliations:** School of Bioengineering & Key Laboratory of Protein Modification and Disease, Liaoning Province, Dalian University of Technology, Dalian, China

**Keywords:** Acetylation, Breast cancer

## Abstract

E74-like ETS transcription factor 5 (ELF5) is involved in a wide spectrum of biological processes, e.g., mammogenesis and tumor progression. We have identified a list of p300-interacting proteins in human breast cancer cells. Among these, ELF5 was found to interact with p300 via acetylation, and the potential acetylation sites were identified as K130, K134, K143, K197, K228, and K245. Furthermore, an ELF5-specific deacetylase, SIRT6, was also identified. Acetylation of ELF5 promoted its ubiquitination and degradation, but was also essential for its antiproliferative effect against breast cancer, as overexpression of wild-type ELF5 and sustained acetylation-mimicking ELF5 mutant could inhibit the expression of its target gene *CCND1*. Taken together, the results demonstrated a novel regulation of ELF5 as well as shedding light on its important role in modulation of breast cancer progression.

## Introduction

Breast cancer is one of the most common malignant tumors in women worldwide, and the incidence of breast cancer is still increasing^1,2^. Approximately 1.3 million females worldwide are diagnosed with breast cancer each year, and ~400,000 people die from the disease. Breast cancer accounts for 25–30% of female malignant tumors in the US and Asia, and it is the second leading cause of cancer death in women^3^. The factors leading to breast cancer oncogenesis and progression are heterogeneous and their clarification requires further study.

E74-like ETS transcription factor 5 (ELF5), also known as epithelium-specific ETS transcription factor 2, belongs to the E26 transformation-specific (ETS) transcription factor family and is characterized by a highly conserved DNA binding domain responsible for recognizing the GGA[AT] core sequence in its target genes^4^. ELF5 is expressed in differentiated keratinocytes and glandular epithelium^5^, and regulates a wide spectrum of biological processes. During pregnancy, ELF5 directs the development of mammary luminal progenitor cells into estrogen receptor-α-negative and progesterone-receptor-negative milk-producing cells^6^. In luminal breast cancer, ELF5 inhibits the transcription of ER, FOXA1, EGFR, MYC, and other proliferation-related genes, resulting in the suppression of estrogen sensitivity^7,8^. Moreover, ELF5 inhibits the transcription of Snail2/Slug and represses epithelial–mesenchymal transition in breast cancer metastasis^9^. In ovarian cancer, overexpression of ELF5 leads to the inhibition of cell viability, invasion, metastasis, and angiogenesis^10,11^. ELF5 binds to AR in androgen receptor (AR)-activated prostate cancer and negatively regulates its transcriptional activity or inhibits SMAD3 activation^12–14^. A sustained increase in ELF5 expression has been demonstrated in endocrine-resistant breast cancers and basal-like subtype breast cancer^5^. ELF5 also drives lung metastasis via the recruitment of GR1^+^ CD11b^+^ myeloid-derived suppressor cells in luminal breast cancer^15^. Hence, the functional role and regulatory mechanism of ELF5 in cancer tumorigenesis and progression are still largely unclear.

The function of a transcription factor is often controlled by post-translational modifications (PTMs). Lysine acetylation is a key form of PTMs that control gene expression, affecting a diverse set of disease signaling pathways in normal cells and during cancer progression^16–18^. Furthermore, acetylation of the nuclear localization signal regions of SKP2 and FoxO1 promotes their nuclear exclusion, resulting in functional changes^19,20^. Moreover, lysine acetylation affects protein functions through diverse mechanisms, and it plays an in increasingly important role in both physiological and pathological processes^16,21–23^. Acetylation of p53 at K120, K164, K320, K370, K372, K373, K381, K382, and K386 sites not only regulates its protein stability, but also influences its DNA-binding ability, while inhibition of p53 acetylation reduces the doxorubicin toxicity in cancer cells^24,25^. Acetylation of EZH2 will lead to its instability and promote the migration and progression of lung cancer^26^. Acetylation of PHF5A occurs upon starvation and modulates stress responses and colorectal tumorigenesis^27^. Acetylation of Ets-1 is the key to chromatin remodeling for miR-192 expression in a transforming growth factor-β-dependent manner^28^. However, there is no study concerning the effects of PTMs on the regulation of ELF5.

In this study, we demonstrated that ELF5 is one of the p300-interacting proteins in human breast cancer cells. It could interact with acetyltransferase p300, resulting in its acetylation at K130, K134, K143, K197, K228, and K245, and this process could be reversed by the deacetylase SIRT6. Acetylation of ELF5 was found to enhance its ability to inhibit the progression of breast cancer via direct regulation of *CCND1* expression.

## Results

### ELF5 is involved in a p300-interaction network

**Fig. 1:**
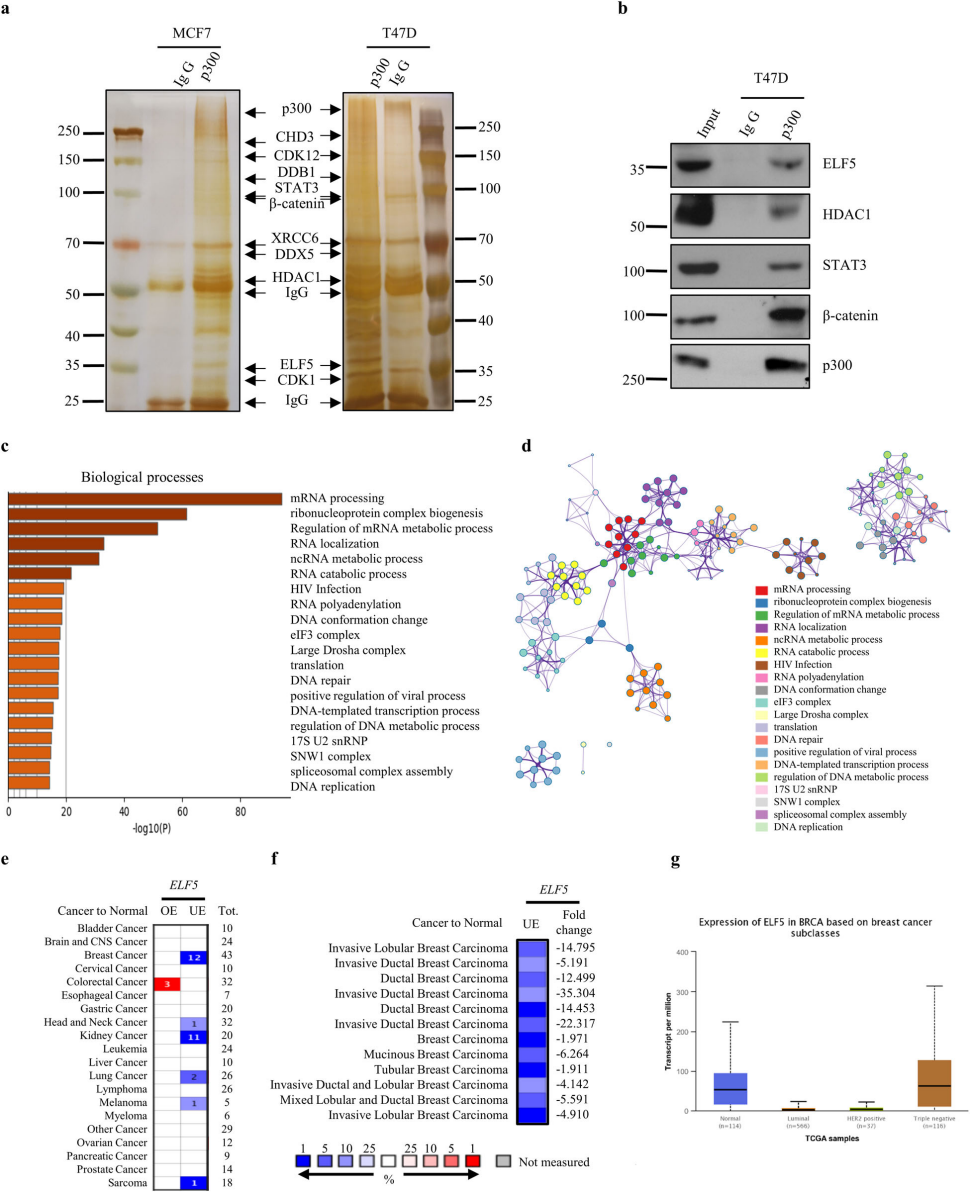
Identification of ELF5 from p300 interaction network. **a** Silver-staining of p300 interacted protein. MCF7 cell lysis and T47D cell lysis were prepared and subjected to affinity purification using anti-p300 antibody or normal IgG. The purified proteins were resolved by SDS-PAGE and visualized by silver-staining. The protein bands were excised and analyzed by mass spectrometry. **b** ELF5 was immunoprecipitated with p300. Whole-cell lysates from T47D cells were subjected to co-immunoprecipitation performed with anti-p300 antibody or normal IgG followed by immunoblotting with performed with the indicated antibodies. Enrichment analysis (**c**) and cluster analysis (**d**) of biological processes according to the results of mass spectrometry. The graphs were downloaded from http://metascape.org/. **e**, **f** Comparison of *ELF5* mRNA expression in healthy and various cancer tissues. Patients datasets were retrieved from the Oncomine database. OE: over-expressed, UE: under-expressed, Tot: total unique analyses. Numbers of significant analyses (*p* < 0.05; fold change >1.5) are shown inside the boxes and total numbers on the right side. Cell color indicates the best gene rank percentile for the analyses (red: over-expressed; blue: under-expressed). *ELF5* mRNA expression as in Fig. 1e. Breast cancer datasets from Fig. 1e were displayed in details in Fig. 1f. **g** The expression of ELF5 in breast cancer based on breast cancer subclasses and data from http://ualcan.path.uab.edu/.

Emerging evidence has revealed that proteins acetylation is involved in various biological events, including gene expression, DNA damage repair, cellular metabolism, cell cycle, signal transduction, and tumor metastasis^16^. p300 is one of the most representative lysine acetyltransferases (KATs) in mammalian cells. Hundreds of p300-acetylated substrates have been identified, e.g., β-catenin, STAT3, and HDAC1, and acetylation is a key form of PTM for their functions^29–32^. To explore the acetylated substrates of p300 in human breast cancer cells, p300-interacting proteins in the MCF7 and T47D cell lysates were immunoprecipitated with an anti-p300 antibody and then identified by mass spectrometry to determine the p300 interactome (Fig. 1a). More than 600 proteins were found to interact with p300 (see Supplementary Table 1). To determine the role of these proteins, we performed a biological process enrichment analysis on Metascape (Metascap, http://metascape.org/)^33^. These p300-interacting proteins were found to be involved in transcription and DNA replication, including mRNA processing, ribonucleoprotein complex biogenesis, DNA conformation change, DNA repair and DNA-templated transcription process (Fig. 1c, d). The biological processes influenced by p300-interacting proteins appear to be consistent with the pathological situation in breast cancer.

Among the p300-interacting proteins identified was ELF5. This was of particular importance considering that ELF5 not only acts as a tumor suppressor by inhibiting the transcription of ER, MYC and Slug in breast cancer but also acts as a carcinogenic factor in basal-like breast cancer cells and endocrine-resistant cells. ELF5 and three other known p300-acetylated proteins (HDAC1, β-catenin and STAT3), were further confirmed by immunoblotting (Fig. 1b). ELF5 was therefore presumed to be a potential acetylated substrate of p300 and the acetylation of ELF5 may be involved in different cellular processes.

### ELF5 is an acetylated protein

**Fig. 2:**
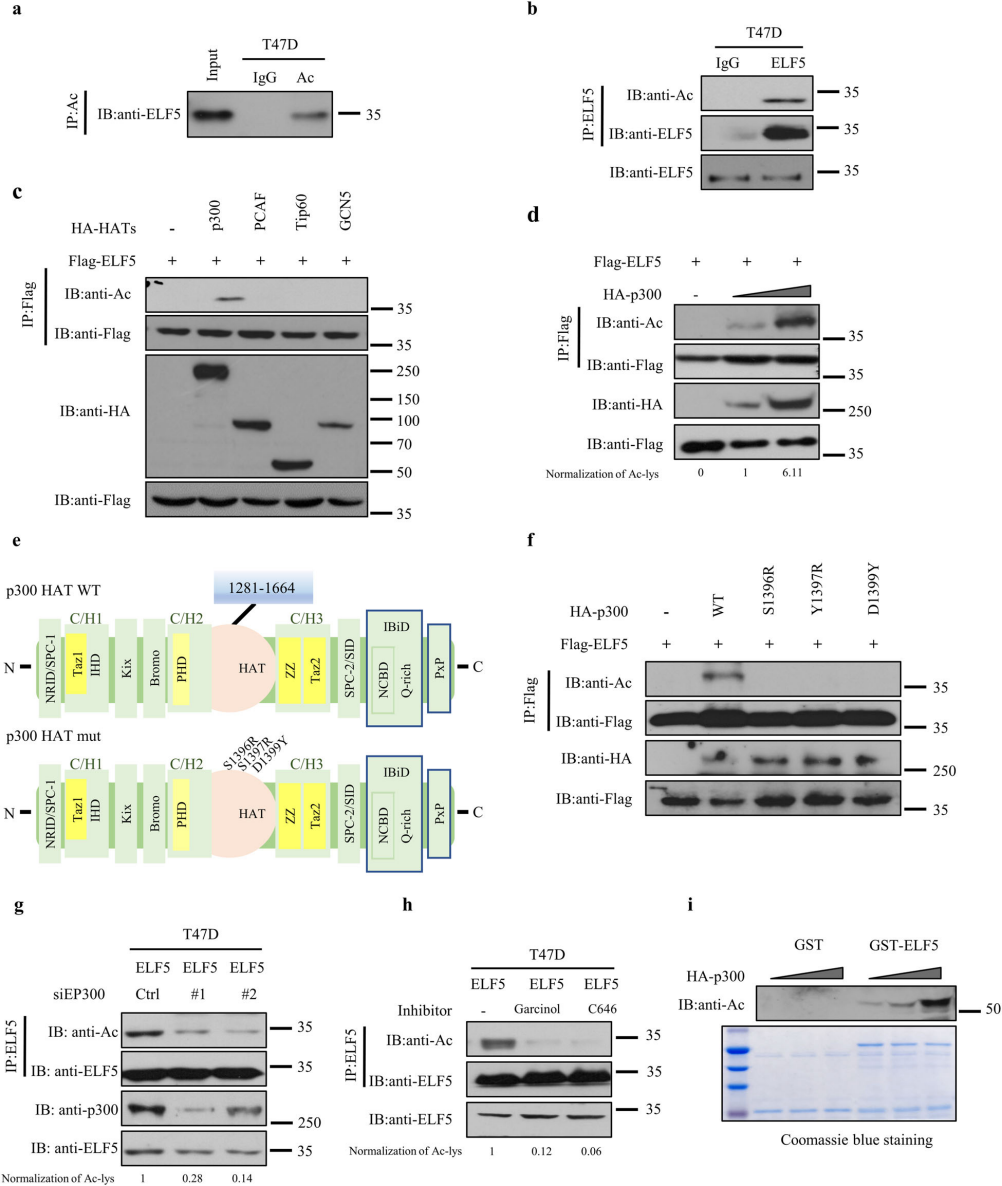
Acetylation of ELF5 by acetylatyltransferase p300. Acetylation of endogenous ELF5. **a** Lysates of T47D cells was immunoprecipitated with an anti-acetylated-lysine (AcK) antibody (Ab) or normal IgG, followed by western blot performed with anti-ELF5 Ab. **b** Lysates of T47D cells was immunoprecipitated with an anti-ELF5 Ab or normal IgG, followed by western blot performed with anti-AcK Ab. **c** p300-mediated acetylation of ELF5. HEK293T cells were co-transfected with Flag-ELF5 and different HA-tagged HATs, HA-p300, HA-PCAF, HA-GCN5, or HA-Tip60, and cell lysate was immunoprecipitated with anti-Flag affinity gel and immunoblotted with the anti-AcK Ab. **d** p300-mediated acetylation of ELF5 in a dose-dependent manner. HEK293T cells were co-transfected with Flag-ELF5 and increasing amounts of HA-p300. After 48 h of transfection, the cell lysates were subjected to co-immunoprecipitation with anti-Flag Ab and immunoblotted with the anti-AcK Ab. **e** Top: A model for full-length p300 showing all the different domains. It was compiled based on several recent analyses, and the catalytic acetyltransferase domain corresponding to residues 1281–1664. Bottom: A model for full-length p300 with single substitutions that inactivate acetyltransferase catalytic activity. **f** Acetylation of ELF5 by p300 depended on its intrinsic KAT activity. HEK293T cells were co-transfected with Flag-ELF5 and p300 or its catalytic mutant, and the cell lysate was subjected to co-immunoprecipitation with anti-Flag affinity gel and immunoblotted with the anti-AcK Ab. **g** Effect of p300 knockdown on the acetylation of ELF5. T47D cells were transfected with siRNA targeting EP300 and then immunoprecipitated with an anti-ELF5 antibody, followed by western blot with anti-AcK Ab. **h** Effect of ELF5 acetylation in the presence of p300 inhibitors. T47D cells were treated without or with Garcinol (10 μM), or C646 (10 μM) for 6 h and then immunoprecipitated with an anti-ELF5 Ab or normal IgG, followed by western blot with anti-AcK Ab. **i** Acetylation of ELF5 by p300 as detected by in vitro acetylation assay. GST-ELF5 fusion protein was affinity-purified and quantified by in vitro acetylation assay. The reaction mixture was subjected to SDS-PAGE and immunoblotted with anti-AcK Ab. Purified GST-ELF5 was examined by Coomassie blue staining.

To investigate the clinical relevance of ELF5 in cancer, publicly available patient datasets were retrieved from the Oncomine database (Oncomine, http://www.oncomine.org/) to examine the differences in ELF5 expression between normal and cancer tissues. ELF5 was found to be downregulated in several types of cancer tissues, especially that of breast cancer (Fig. 1e, f). Furthermore, a higher expression of ELF5 was detected in triple-negative breast cancer than in luminal and her-2 positive breast cancer (Fig. 1g). Previous studies have shown that methylation of the promoter of ELF5 is the main reason for the decline in its expression^34^. ELF5 expression is also upregulated in basal-like breast cancer and endocrine-resistant breast cancer^5,7^. Considering the different levels of ELF5 expression in breast cancer molecular subtypes, we speculated that acetylation might be involved in the modulation of ELF5 functions. Sequence analysis of ELF5 revealed 15 conserved lysine residues among the vertebrate orthologs (Supplementary Fig. 1), suggesting that ELF5 could be a potential target for PTM by lysine acetylation. Subsequent co-immunoprecipitation assay confirmed the acetylation of exogenously expressed ELF5 (Supplementary Fig. 2a). To investigate the acetylation of endogenous ELF5, the expression of endogenous ELF5 was evaluated in MCF7, T47D, HEK293T, and Hela cells. ELF5 was found to be highly expressed in T47D and HEK293T cells, but only slightly expressed in MCF7 cells, while no expression was detected in Hela cells (Data not shown). Subsequent immunoprecipitation performed with T47D cells and an anti-acetylated-lysine antibody followed by probing with anti-ELF5 antibody revealed a band corresponding to ELF5 (Fig. 2a). Similar immunoprecipitation followed by probing with anti-acetylated-lysine antibody also revealed the corresponding ELF5 band (Fig. 2b). Besides, immunoprecipitation carried out using MCF7 and HEK293T cells also yielded the same result (Supplementary Fig. 2b–d). These experiments clearly demonstrated that ELF5 is subject to acetylation in cells.

### ELF5 is acetylated by acetyltransferase p300

The data from immunoblot analysis strongly suggested the acetylation of ELF5 by p300, but it did not rule out the possibility that other acetyltransferase might be responsible for the acetylation of ELF5. To clarify this, Flag-ELF5 was co-expressed with each of the four major lysine acetyltransferases (KATs), p300, PCAF, Tip60, and GCN5, in HEK293T cells, and the cell lysates were subjected to affinity purification with anti-Flag affinity gel. Acetylation of ELF5 was detected when ELF5 was co-expressed with p300, but was not detected when other acetyltransferases were used instead, including PCAF, Tip60, and GCN5 (Fig. 2c). Considering that a high endogenous level of ELF5 would compete with the exogenously expressed ELF5, similar experiments were performed in Hela cells to avoid missing other potential acetyltransferases. The results confirmed that only p300 could acetylate ELF5 (Supplementary Fig. 3a). Overexpression of both ELF5 and p300 increased the extent of ELF5 acetylation in a dose-dependent manner (Fig. 2d). To examine whether the acetylation of ELF5 by p300 might involve the intrinsic KAT activity, three catalytically inactive mutants (S1396R, Y1397R, and D1399Y) of p300 were tested (Fig. 2e)^19,35^. As anticipated, ELF5 acetylation was not detected when these p300 mutants were co-expressed with ELF5 (Fig. 2f), implying that p300 acetyltransferase activity was required for the acetylation of ELF5. Furthermore, when p300 was knocked down, there was a significant reduction of ELF5 acetylation (Fig. 2g). ELF5 acetylation was inhibited to a large extent in the presence of Garcinol^19^ and C646^36^, both inhibitor of p300 (Fig. 2h). ELF5 acetylation gradually increased as more p300 was expressed in the in vitro acetylation assays (Fig. 2i). The data, therefore, revealed for the first time, that ELF5 is a protein subject to post-translational modification via p300-mediated acetylation.

The interaction between ELF5 and p300 was further demonstrated by co co-immunoprecipitation. When HEK293T cells were co-transfected with GFP-tagged ELF5 and HA-tagged p300, both proteins were co-immunoprecipitated using either anti-GFP or anti-HA antibody (Supplementary Fig. 3b, c), confirming that ectopically expressed ELF5 was indeed associated with p300. Furthermore, in T47D cells, endogenous p300 was also co-immunoprecipitated with endogenous ELF5 (Supplementary Fig. 3d). The interaction of ELF5 with p300 was further confirmed by GST pull-down assay, whereby GST-ELF5 was shown to interact directly with the overexpressed HA-p300 in HEK293T cells (Supplementary Fig. 3e). Finally, immunofluorescence staining assay showed that both ELF5 and p300 were localized in the nucleus of T47D cells (Supplementary Fig. 3f). Taken together, we demonstrated for the first time that ELF5 could be acetylated by p300 both in vitro and in vivo.

### ELF5 is acetylated at K130, K134, K143, K197, K228, and K245 by p300

**Fig. 3:**
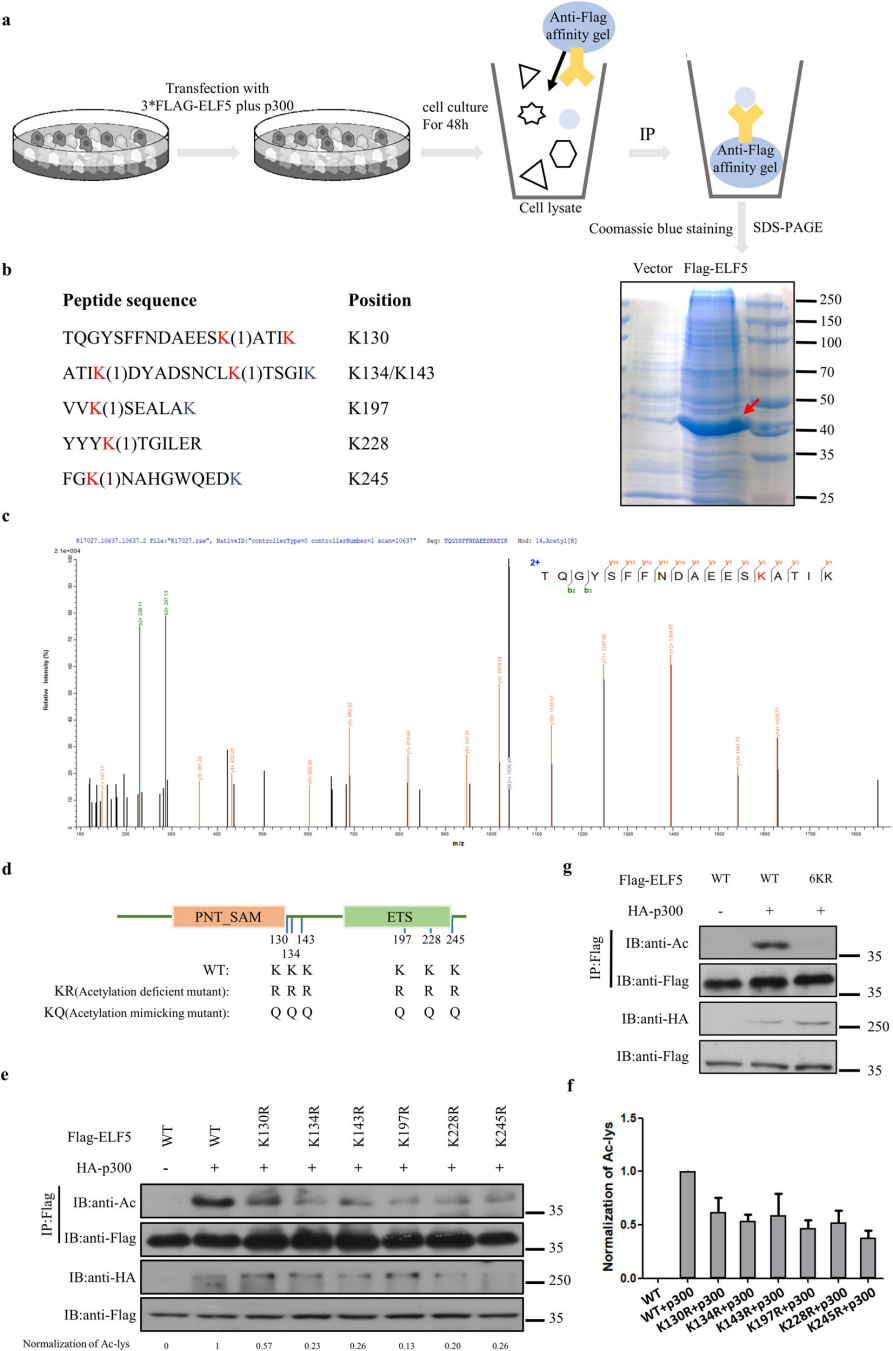
ELF5 is acetylated at K130, K134, K143, K197, K228 and K245. **a** Diagram outlining the steps involved in the preparation of sample for mass spectrometry (MS) to identify the acetylation lysine sites of ELF5. **b** Mass spectrometry identification of ELF5 acetylation sites in HEK293T cells transfected with expression plasmids for Flag-ELF5 and HA-p300. **c** MS analysis of the ELF5-derived peptides containing acetylated K130. **d** Diagram showing the acetylation sites of ELF5 that were mutated. **e** Effects of lysine to arginine at putative acetylated lysine residues on the acetylation of ELF5. Hela cells co-transfected with Flag-ELF5 WT or mutant Flag-ELF5 plus HA-p300 or not, cell lysis was immunoprecipitated with anti-Flag affinity gel and immunoblotted with the anti-AcK Ab. **f** Quantitative analyses of the acetylation of ELF5 WT and mutants. **g** The acetylation of ELF5 6KR was abolished. HEK293T cells co-transfected Flag-ELF5 WT or Flag-ELF5 6KR without and with HA-p300. Cell lysis was immunoprecipitated with anti-Flag affinity gel and immunoblotted with the anti-AcK Ab.

Given that ELF5 could be acetylated by p300 both in vivo and in vitro, it became important to identify the acetylation sites of ELF5. Acetylated ELF5 purified by immunoprecipitation was resolved by SDS-PAGE followed by Coomassie blue staining and mass spectrometry analysis (Fig. 3a). Of the 15 lysine residues in ELF5, 6 were found to be acetylated, and they were Lys-130, Lys-134, Lys-143, Lys-197, Lys-228, and Lys-245, whereas the rest were not acetylated (Fig. 3b, c and Supplementary Fig. 4). These six lysine residues were found to be highly conserved by sequence alignment (Supplementary Fig. 1). To demonstrate the importance of these lysine residues in ELF5 acetylation, lysine to arginine (mimics of acetylation deficiency) substitution was used to verify each acetylation site. The result revealed a weak attenuation of ELF5 acetylation in the single mutants (Fig. 3d, cumulative data from repeat experiments with a quantitative difference are shown in Fig. 3f), while substitution of all the six lysine residues with arginine (6KR) completely abrogated the acetylation of ELF5 (Fig. 3g). Taken together, our data suggested that ELF5 is subject to extensive acetylation mediated by p300.

### SIRT6 specifically interacts with and deacetylates ELF5

**Fig. 4:**
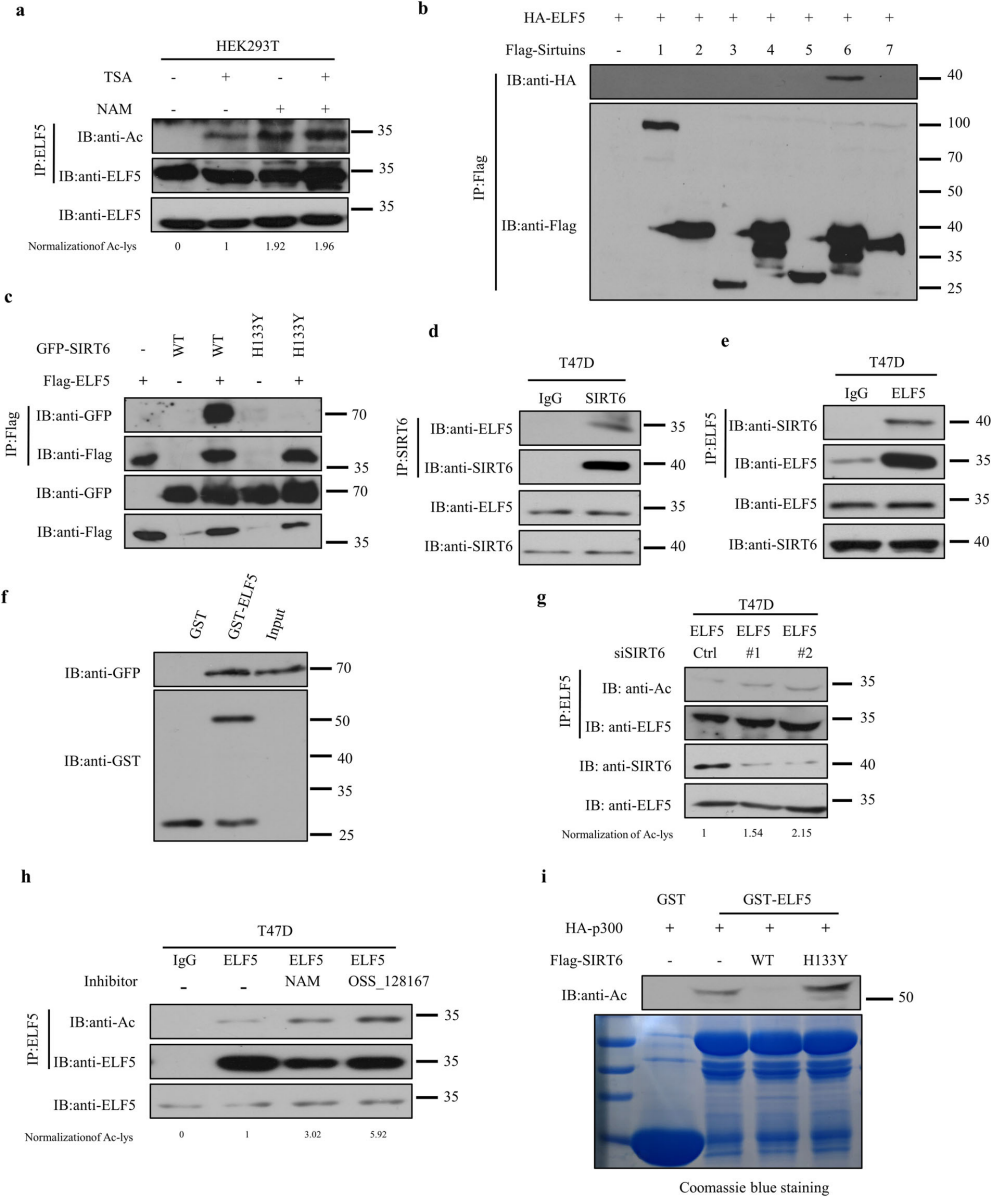
SIRT6 specifically interacts with and deacetylates ELF5. **a** Deacetylase inhibitors increased the acetylation level of ELF5. HEK293T cells were treated without and with TSA or NAM, or both TSA and NAM, and cell extract was immunoprecipitated with an anti-ELF5 antibody, followed by western blot with the anti-AcK Ab. **b** Exogenous ELF5 interacted with SIRT6. HA-ELF5 was overexpressed with different Flag-Sirtuin in HEK293T cells, followed by immunoprecipitation with anti-Flag affinity gel and western blot with anti-HA Ab. **c** Exogenous ELF5 bound to wild type SIRT6. Flag-ELF5 was co-expressed with GFP-SIRT6 or the catalytic mutant H133Y, and the cell extract was subjected to affinity purification with anti-Flag affinity gel, followed by Western blot with the indicated antibodies. Endogenous interaction between ELF5 and SIRT6 in T47D cells as detected by IP. T47D cells were subjected to immunoprecipitation with anti-SIRT6 Ab or normal IgG followed by Western blot with anti-ELF5 Ab (**d**). T47D cells were subjected to immunoprecipitation with anti-ELF5 Ab or normal IgG followed by Western blot with anti-SIRT6 Ab (**e**). **f** Interaction between ELF5 and SIRT6 as demonstrated by GST-pulldown assay. Extract of HEK293T cells overexpressing GFP-SIRT6 was subjected to GST-pulldown assays with GST or GST- ELF5. **g** Effects of SIRT6 knockdown on the acetylation of ELF5. T47D cells were transfected with siRNA targeting SIRT6 and then immunoprecipitated with an anti-ELF5 Ab, followed by western blot with anti-AcK Ab. **h** Effect of ELF5 acetylation in the presence of SIRT6 inhibitors. T47D cells were treated without or with NAM (5 mM), or OSS_128167 (200 μM) for 6 h and then immunoprecipitated with an anti-ELF5 Ab or normal IgG, followed by western blot with anti-AcK Ab. **i** Deacetylation of ELF5 by SIRT6 as demonstrated by in vitro deacetylation assay. GST-ELF5 was affinity-purified and measured by in vitro deacetylation assays. Pre-acetylated GST-ELF5 was incubated with immunoprecipitated Flag-SIRT6 WT or Flag-SIRT6 H133Y in the presence of NAD^+^. The reaction mixtures were subjected to SDS-PAGE and immunoblotted with anti-AcK Ab. The purified GST fusion proteins were examined by Coomassie blue staining.

Acetylation is a dynamic modification process catalyzed by specific acetyltransferase(s) and reversed by specific deacetylase(s)^37^. Thus, identifying the deacetylase responsible for reversing the acetylation of ELF5 would seem logical. Lysine deacetylases are divided into two categories: classical Zn^2+^-dependent histone deacetylases (HDACs) and NAD^+^-dependent sirtuin deacetylases, which can be inhibited by TSA and nicotinamide (NAM), respectively. A low-intensity band corresponded to acetylated ELF5 was detected when TSA was added to HEK293T cells before the cell lysis step, but a more intense band with similar molecular weight was seen when the cells were treated with NAM before the lysis step (Fig. 4a). The level of ELF5 acetylation was significantly raised in the presence of TSA and NAM (Fig. 4a). By considering the important role of the sirtuin family of deacetylases^38,39^ and the fact that treatment of NAM increased the acetylation of ELF5 considerably, we reasoned that the sirtuin family deacetylases might be the most important negative regulators of ELF5 acetylation. To test this hypothesis, we co-expressed HA-ELF5 with the Flag-tagged sirtuin members in HEK293T cells. Among the SIRT1-7 deacetylases, only SIRT6 was found to specifically interact with ELF5 (Fig. 4b). To confirm the interaction between ELF5 and SIRT6, another co-immunoprecipitation assay was conducted for ELF5 and wild-type SIRT6 or the catalytically inactive SIRT6 mutant, H133Y. ELF5 was found to interact with the wild-type SIRT6 but not the mutant (Fig. 4c). Furthermore, the interaction between SIRT6 and ELF5 was validated at the endogenous level and by in vitro pull-down assays conducted with the recombinant GST-ELF5 (Fig. 4d–f). Conversely, the acetylation of endogenous ELF5 in T47D cells was drastically increased when SIRT6 was inhibited or knocked down (Fig. 4g, h). At the same time, the acetylation of ELF5 was reduced significantly by SIRT6, not the catalytically inactive SIRT6 H133Y, in the in vitro deacetylation assay (Fig. 4i). Our data, therefore, revealed SIRT6 as an ELF5-interacting protein that could deacetylate ELF5, demonstrating that acetylation of ELF5 could be a dynamic and tightly regulated process in living cells.

### Acetylation promotes ubiquitin-proteasome degradation of ELF5

**Fig. 5:**
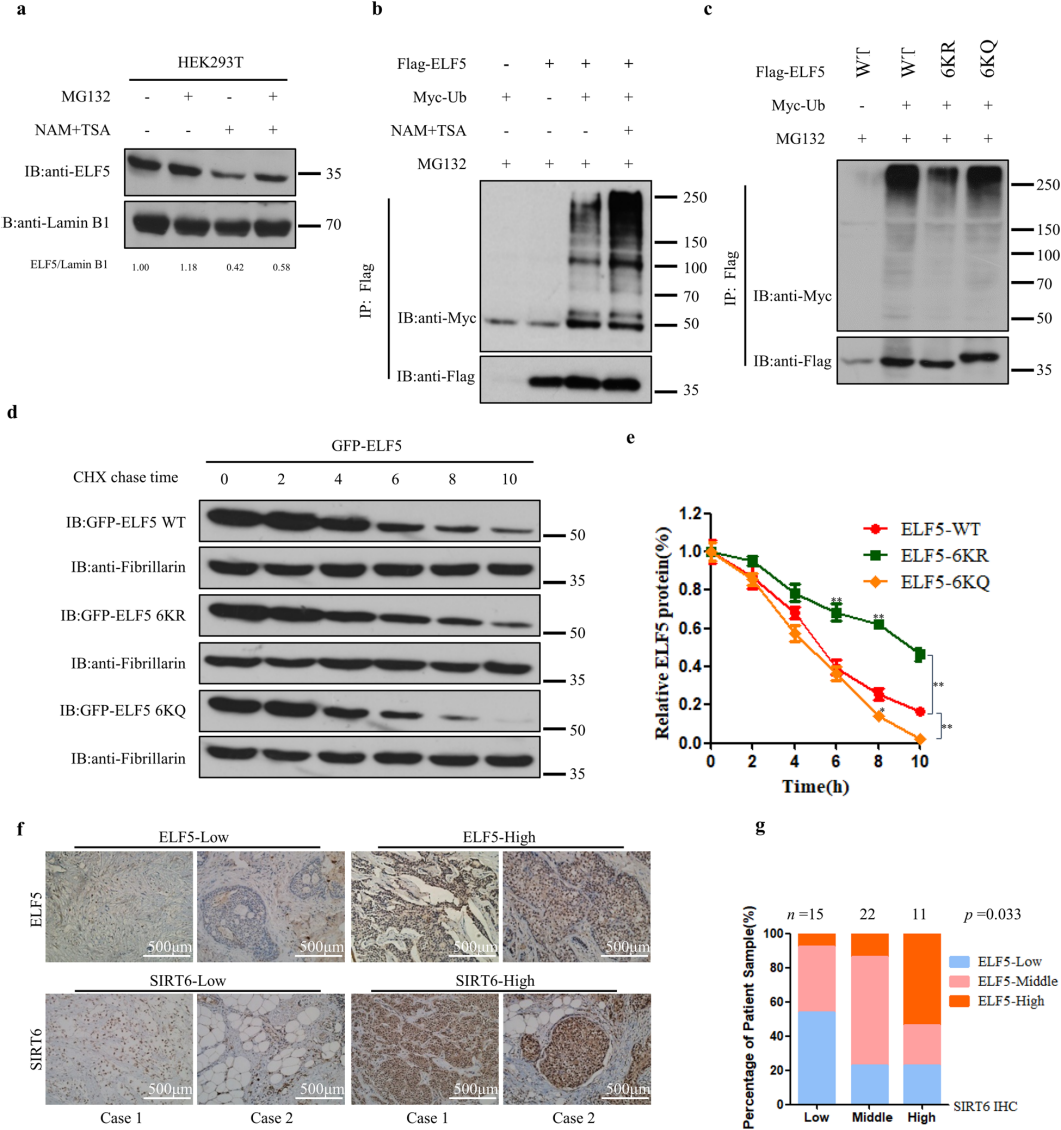
Acetylation promotes the proteasome-mediated degradation of ELF5. **a** Inhibition of deacetylation and protein synthesis on the protein level of ELF5. HEK293T cells were treated without and with 2 μM TSA and 5 mM NAM in the absence or presence of 20 μM MG132. Endogenous ELF5 was probed by anti-ELF5 Ab. **b** NAM- and TSA-mediated regulation of the ELF5 ubiquitination. Ubiquitination of Flag-ELF5 expressed in HEK293T cells the absence and presence of NAM + TSA with MG132. **c** Quantitation of the ubiquitination levels of Flag-ELF5 WT, Flag-ELF5 6KR, and Flag-ELF5 6KQ in HEK293T cells. **d**, **e** Half-lives of ELF5 and mutants. HEK293T cells expressing GFP-ELF5-WT, GFP-ELF5-6KR, or GFP-ELF5-6KQ were treated with 50 μM Cycloheximide, and the expression levels of these proteins were analyzed by western blot with anti-GFP Ab. The graph shown represents the ELF5 half-lives in Fig. 5e. Data are the means ± SDs from three determinations. *p < 0.05; **p < 0.01. **f**, **g** The expression of ELF5 and SIRT6 in human breast cancer tissues. Representative images tissue samples from breast cancer patients showing the expression levels of ELF5 and SIRT6 as assessed by immunohistochemistry. Both ELF5 and SIRT6 levels were classified as low, medium, or high based on the intensities of their IHC staining, and the percentages of patients classified in each category are depicted in the histogram in Fig. 5g.

To elucidate the effect that acetylation might have on the stability of ELF5, HEK293T cells were treated with MG132 (a proteasomal degradation inhibitor) or NAM or both and then the level of endogenous ELF5 was measured. The level of endogenous ELF5 was increased by 18% upon treatment with MG132, whereas the protein levels were decreased by almost 58% with the treatment of NAM. When both MG132 and NAM were added to the cell culture medium, the steady-state levels of cellular ELF5 was partially restored (Fig. 5a). The destabilization effect of NAM treatment on ELF5 was suppressed by the action of MG132. Similar to this observation, the addition of p300 also reduced the protein level of ELF5, but MG132 could reverse this effect of p300 on the protein level of ELF5 to some extent (Supplementary Fig. 5a). In contrast, when ELF5 was co-transfected with SIRT6, the stability of ELF5 was enhanced upon treatment with MG132 (Supplementary Fig. 5b). These results indicated that the decrease in ELF5 level promoted by its acetylation might be mediated by the ubiquitin-proteasome pathway. When Flag-ELF5 was co-expressed with Myc-ubiquitin, active ELF5 ubiquitination was detected, while inhibition of deacetylases significantly increased ELF5 ubiquitination in the presence of MG132 (Fig. 5b). Furthermore, p300 also increased the ubiquitination of ELF5 and inhibition of deacetylases had an enhancing effect on the ubiquitination of ELF5 (Supplementary Fig. 5c). When ELF5-6KR plus Myc-Ub were co-expressed in HEK293T cells, the mutant ELF5 protein exhibited a lower basal level of ubiquitination than its wild-type counterpart and the ELF5-6KQ mutant, an acetylation mimic mutant (Fig. 5c), implying that the ubiquitination of ELF5 might depend on its acetylation. Cycloheximide chase assay showed that 10 h after the addition of CHX, approximately 80% of ELF5-WT, 55% of ELF5-6KR, and 100% of ELF5-6KQ were degraded. The half-lives of ELF5-WT, ELF5-6KR and ELF5-6KQ were determined to be 5 h, 9 h, and 4 h, respectively (Fig. 5d, e). These results signified that increasing the acetylation of ELF5 could lead to its ELF5 instability. In support of this view, a positive correlation between ELF5 and SIRT6 was found in 48 breast cancer clinical samples by immunohistochemical staining (Fig. 5f, g).

### ELF5 acetylation inhibit cancer cell proliferation and tumorigenesis

**Fig. 6:**
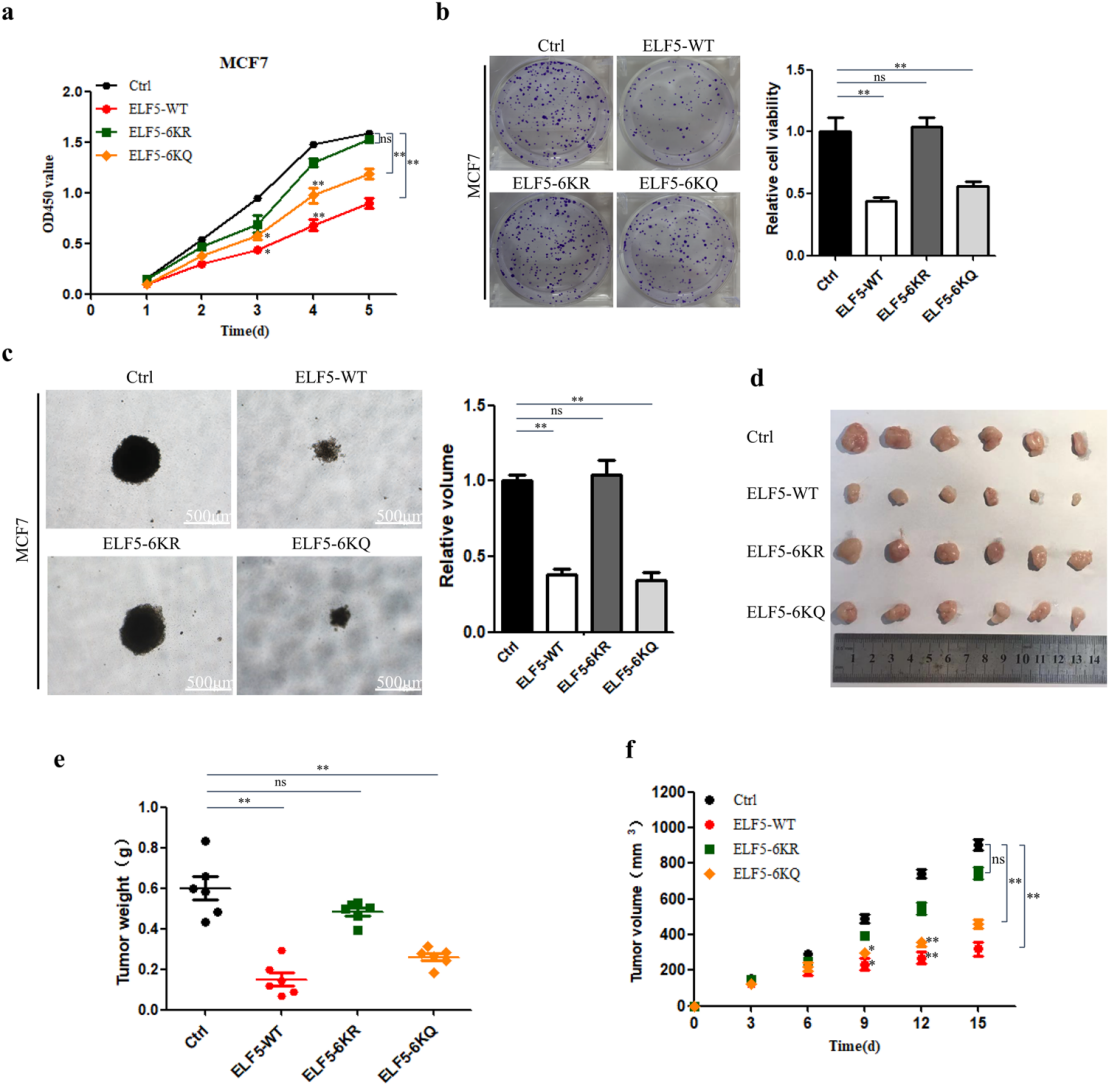
Inhibition of breast cancer cell proliferation and tumorigenesis by ELF5 acetylation. **a** Growth curves of MCF7 cells that stably expressed GFP-ELF5 WT, GFP-ELF5 6KR, GFP-ELF5-6KQ as measured by CCK8 assay. Control cells were transfected with just the pEGFP-C1. Data are the means ± SDs from three determinations. *p < 0.05; **p < 0.01; ns, no significance. **b** Colony formation of MCF7 cells that stably expressed GFP-ELF5 WT, GFP-ELF5 6KR, GFP-ELF5-6KQ as measured by crystal violet staining assay. Control MCF7 cells were transfected with just the vector pEGFP-C1. Data are the means ± SDs from three determinations. *p < 0.05; **p < 0.01; ns, no significance. **c** Anchorage-independent growth activity of MCF7 cells as measured by soft-agar colony culture assay. Data are the means ± SDs from three determinations. *p < 0.05; **p < 0.01; ns, no significance. **d** Image showing the tumor formed in xenograft mice. **e**, **f** Weight and growth of tumor induced in mice by subcutaneously implanted MCF7 cells (5 × 10^6^ cells injected per mice) that stably expressed GFP-ELF5 WT, GFP-ELF5 6KR, GFP-ELF5-6KQ. Control MCF7 cells were transfected with just the vector pEGFP-C1. Data are the means ± SDs from three determinations. *p < 0.05; **p < 0.01; ns, no significance.

To further test the physiological function of ELF5 acetylation in breast cancer cells, we investigated the effect of ELF5 acetylation on cancer cell proliferation and tumor growth in mice. First, MCF7 and Hela cells that overexpressed ELF5-WT, ELF5-6KR, and ELF5-6KQ were subjected to CCK8 assay to test the effect of ELF5 acetylation on cell proliferation. Overexpression of ELF5-WT in MCF7 cells caused a significant decrease in the proliferation rate compared with the control cells (transfected with just empty vector), consistent with previous studies^7^. Cells expressing the acetylation-deficient mutant ELF5-6KR showed no reduction in cell proliferation. Furthermore, cells expressing the acetylation-mimic mutant ELF5-6KQ exhibited reduced proliferation to some extent compared with the control cells (Fig. 6a). Similar results were also observed for Hela cells (Data not shown). Colony formation and soft-agar colony culture assays revealed a marked decrease in colony formation and volume for cells that expressed wild-type ELF5-WT. Cells that overexpressed ELF5-6KR displayed more vigorous growth and had bigger colonies than those that overexpressed ELF5-6KQ (Fig. 6b, c). ELF-6KQ could function as a wild type, consistent with the model of heme oxygenase-1 acetylation^22^. We speculated that in MCF-7 cells, wild-type ELF5 might be highly acetylated, as in the case of the mutant ELF5-6KQ, and therefore, the extent of inhibition of cell proliferation might be similar when MCF-7 cells overexpress wild-type ELF5-WT or ELF-6KQ. MCF7 cells stably transfected with ELF5-WT, ELF5-6KR, ELF5-6KQ, or the control vector were inoculated into the flanks of nude mice to perform the xenograft assay to further test the tumor suppressing role of ELF5 acetylation. All mice injected with MCF7 cells developed tumors within 4 weeks (Fig. 6d–f). Compared with the control cells, the cells that expressed ELF5-WT formed the smallest tumors and at the slowest rate. Furthermore, the cells that expressed ELF5-6KQ displayed a slower tumor growth rate and a smaller tumor volume than the control cells, similar to the cells that expressed ELF5-WT. However, the tumor volume and growth rate of the cells expressing ELF5-6KR were consistent with the control group. Thus, these results strongly indicated that in mice, the acetylation of ELF5 could play a tumor inhibitory role by suppressing the proliferation of breast cancer cells and breast carcinogenesis.

### Acetylation of ELF5 inhibit *CCND1* transcription

**Fig. 7:**
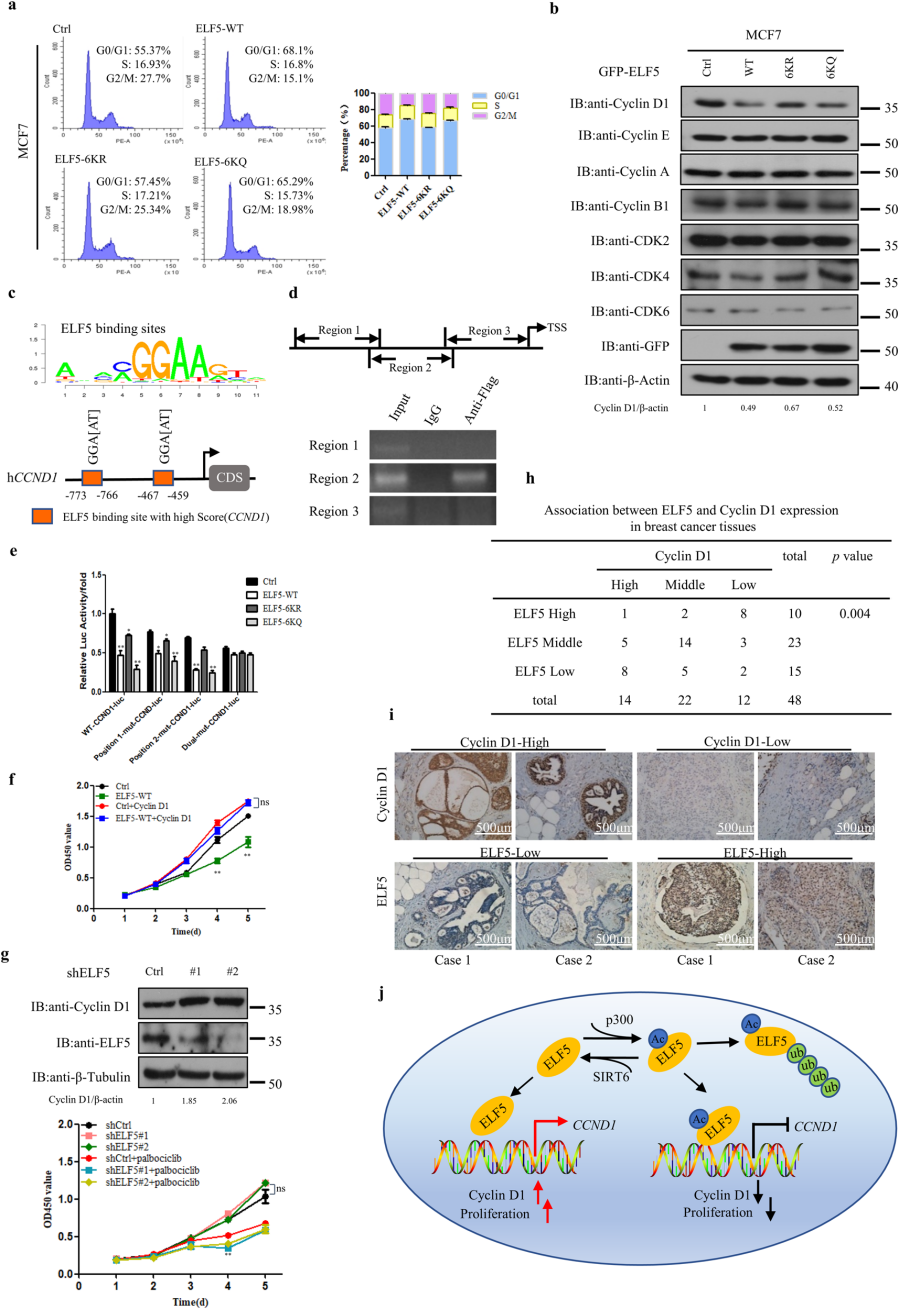
Inhibition of *CCND1* transcription by ELF5 acetylation. **a** Flow cytometry analysis of the cell cycle showing the distribution of MCF7 cells stably transfected with GFP-ELF5 WT, GFP-ELF5 6KR, GFP-ELF5-6KQ or control vector. **b** Western-blot detection of cyclins and CDKs expression in MCF7 cells that overexpressed with GFP-ELF5 WT, GFP-ELF5 6KR, GFP-ELF5-6KQ or the control vector. **c** Schematic representation of the conserved ELF5-binding motif and on *CCND1* potential promoter region. **d** Regions of *CCND1* promoter physically associated with ELF5 as analyzed by ChIP assay. Top: schematic illustration of the PCR-amplified fragments of *CCND1* promoter; bottom: ChIP assay was carried out using anti-Flag antibody to screen for ELF5-bound *CCND1* promoter regions in HEK293T cells. **e** Effect of mutations within the ELF5-binding site of *CCND1* promoter on its activity. *CCND1* promoter with a site-specific mutation within the ELF5-binding site was fused to a luciferase reporter gene. MCF-7 cells transfected with such construct and either Flag-ELF5 WT, Flag-ELF5 6KR, Flag-ELF5-6KQ or the vector pEGFP-C1 were subjected to luciferase activity assay. Data are the means ± SDs from three determinations. *p < 0.05; **p < 0.01. **f** Growth curves of MCF7 cells that stably overexpressed with GFP-ELF5 without or with HA-cyclin D1 overexpression as measured by CCK8 assay. *p < 0.05; **p < 0.01; ns, no significance, compared with cells transfected with just the pEGFP-C1 vector. **g** Top: T47D cells expressing a shCtrl or shELF5 were analyzed for Cyclin D1 protein level. Bottom: Growth curves of T47D cells expressing a shCtrl or shELF5 without or with palbociclib treatment as measured by CCK8 assay. *p < 0.05; **p < 0.01; ns, no significance. **h** Correlation between Cyclin D1 and ELF5 expression in breast cancer tissues. Two-side *χ*2-test was used for statistical analysis. **i** Representative images of tissue samples from breast cancer patients showing the expression levels of ELF5 and Cyclin D1 as assessed by immunohistochemistry. Both ELF5 and Cyclin D1 levels were classified as low, medium, or high based on the intensities of the IHC staining. **j** Proposed model depicting the regulation of ELF5 by acetylation. In this model, ELF5 is acetylated by acetyltransferase p300 and deacetylated by deacetylase SIRT6. Acetylation of ELF5 increases its capacity to suppress breast cancer proliferation by suppressing the transcription of its target gene *CCND1*.

To clarify the molecular mechanism underlying the regulatory effect of ELF5 acetylation on cell proliferation and tumorigenesis, changes in the cell cycle were observed via flow cytometry. Overexpression of ELF5-WT in MCF7 cells elicited a substantial increase in the number of cells in the G1 phase and a concomitant reduction in the number of cells in the G2/M phase (Fig. 7a). Changes in the cell cycle observed were similar to those reported in previous researches^40,41^. Considering that cyclins and CDKs are closely associated with the cell cycle progression^42–45^, specific cell cycle markers were detected and compared by Western blotting. No obvious difference in the levels of cyclin E, cyclin A, cyclin B1, and CDKs was observed among the cells that overexpressed ELF5-WT, ELF5-6KR, ELF5-6KQ, and the control cells, but a consistent downregulation of cyclin D1 was evident in the cells that overexpressed ELF5-WT and ELF5-6KQ compared with the control and those that overexpressed ELF5-6KR (Fig. 7b). These changes of cyclin D1 were also confirmed at the transcriptional level (Supplementary Fig. 5a).

ELF5 is an ETS transcription factor that can bind to DNA sequences containing the consensus nucleotide core sequence GGA[AT]. Previous studies have confirmed that Cyclin D1 is regulated by members of the ETS family, such as c-ETS-2 and ETV4^46,47^. In 50% of breast cancer patients, cyclin D1 is highly expressed and cyclin D1 is an important marker of cell proliferation^44,48^. To investigate whether ELF5 might transcriptionally regulate cyclin D1 expression, bioinformatic analysis was performed to identify the putative ELF5-binding sites in the promoter of the human *CCND1* gene. Two putative ELF5 sites were identified in the promoter, at positions −773bp and −467bp from the transcription start site (Fig. 7c). This result was further confirmed by ChIP assay conducted with HEK293T cells. ELF5-WT was enriched in region 2 (−821, −415bp) of the *CCND1* promoter, but not in region 1 (−1187, −822bp) and region 3 (−414, +1 bp) (Fig. 7d). When the two conservative motifs were mutated from GGAA to GCTA, the transcriptional inhibitory effect of ELF5 on *CCND1* disappeared as shown by reporter gene assay (Fig. 7e). These results suggested that ELF5 inhibited the expression of *CCND1* by interacting with the sites located within regions −773 to −766 and −467 to −459 of the *CCND1* promoter. The inhibitory effect of ELF5 on tumor proliferation was abolished in a *CCND1* rescue experiment or by overexpression of Cyclin D1, suggesting that the function of ELF5 might be *CCND1*-dependent (Fig. 7f, Supplementary Fig. 6d, e). CDK4/6 inhibitors such as palbociclib, abemaciclib, and ribociclib have been clinically approved for breast cancer treatment. Silencing the expression of ELF5 in T47D cells increased both the expression of Cyclin D1 and the inhibition effect of the CDK4/6 inhibitors (Fig. 7g, Supplementary Fig. 6f, g), suggesting that ELF5 expression can be used to stratify patients that may be responsive to CDK4/6 inhibitors. Immunohistochemical staining of 48 breast cancer tissue samples shed further light on the pathological relevance of ELF5 in the regulation of Cyclin D1 expression in breast cancer. Lower Cyclin D1 level was found to correspond to higher ELF5 expression in tumor tissues (Fig. 7h, i). Statistical analysis of the stained images indicated a negative correlation between ELF5 and Cyclin D1 levels. Therefore, Cyclin D1 could act as an important downstream effector to mediate the suppressive role of ELF5 in breast cancer progression.

## Discussion

Protein acetylation is involved in numerous cellular processes, such as chromatin remodeling, cell cycle, transcriptional regulation, DNA repair, cell metabolism and autophagy. Moreover, the widespread nature of protein acetylation and the large number of identified acetylated proteins have raised doubts about the role of individual KATs and their importance in regulating different cellular functions. Through LC-MS/MS analysis, we found a large number of p300-interacting proteins and ELF5 was among them (Fig. 1a, b). ELF5 was identified as an acetylated protein in breast cancer cells and later shown to be subject to regulation by the acetyltransferases p300 and the deacetylase SIRT6 (Fig. 7j). In HEK293T cells, the stability of ELF5 was decreased by the overexpression of p300 in the presence of a deacetylase inhibitor (Fig. 5a and Supplementary Fig. 5a). The result demonstrated the occurrence of crosstalk between acetylation and ubiquitination of ELF5. Data obtained from CCK8 assay, colony formation, and xenograft mouse model indicated that acetylation of ELF5 is vital for its inhibition of cell proliferation in breast cancer. Mechanistically, the expression of Cyclin D1 was transcriptionally inhibited by ELF5 via two GGA[AT] conservative motifs, which resulted in cell arrest at the G1 phase of the cell cycle (Fig. 7c–f). Thus, these results presented a previously undefined mechanism of ELF5 acetylation in the control of breast cancer cell growth and tumor progression.

ELF5 acetylation might be involved in the regulation of gene transcription. Most of the canonical lysine acetyltransferases are localized in the nucleus where they can influence the transcriptional activity of the acetylated proteins by functioning as transcription co-activators. For example, the acetylation of p53 controls the activation of p53-regulated genes in response to cellular stresses^49^. Similarly, the acetylation of ERα at lysine 266 and 268 enhances its DNA binding and transactivation activities^50^. ELF5 is a member of the ETS transcription factor family, and it binds to the GGA[AT] ETS motif found in the promoter regions of Slug and mCcnd2^9,51^. The data from ChIP and luciferase reporter gene assays demonstrated the direct inhibition of *CCND1* expression by ELF5 through the ETS motif of the *CCND1* promoter (Fig. 7c–e). The inhibitory effect of ELF5 in breast cancer was abolished when Cyclin D1 was overexpressed or *CCND1* was knockdown (Fig. 7g and Supplementary Fig. 6c). A number of ELF5 target genes have been identified by ChIP assay in a previous study, and the *CCND1* gene was not among those identified, although the expression of Cyclin D1 was found to be downregulated by western blot^7^. Considering that ELF5 can directly inhibit ERα transcription and *CCND1* is a downstream target gene of ERα, the down-regulation of Cyclin D1 observed in this study might be related to the function of ERα. However, a study carried out by Catherine and coworker found that Cyclin D1 is downregulated by ELF5 in T47D cells and MDA-MB-231 cells^5^. This means that the inhibitory effect of ELF5 on *CCND1* is ERα-independent. Thus, according to our data, *CCND1* could be a target gene of ELF5, indicating that the execution of a tumor suppressor via *CCND1* may be another action mechanism of ELF5.

The acetylation of transcription factors may exert either a stimulatory or inhibitory effect on the transcription of target genes via a variety of mechanisms. Acetylation can enhance the stability of SMAD7 and p53^49,52,53^, but disrupts the stability of DNMT1 and PEPCK1^54,55^. At present, other relevant factors contributing to the stability of ELF5 have not been studied. The predicted ubiquitination sites of ELF5 are available for sequence alignment and download on the website: Predictor of ubiquitination sites, UbPred, http://www.ubpred.org/. K130, K134, K143, and K148 with medium confidence (from 0.71 to 0.83) were considered to be the most likely ubiquitination sites. Among the six acetylated lysines in ELF5, K130, K134, and K143 might also be modified by ubiquitination. The underlying molecular mechanisms could either help to recruit potential E3 ubiquitin-protein ligase or allow ELF5 to interact with proteins that shield it from being recognized by deubiquitinase or enzymatic dissociation, resulting in reduced ELF5 protein stability.

A crucial question is whether the degradation of ELF5 and the activation of its transcription induced by ELF5 might be interconnected. The results we obtained were not consistent with conventional view that decreased protein stability will lead to inhibition of transcriptional activity. However, the enhanced transcriptional activity of several important proteins is accompanied by a decrease in protein stability. Acetylation of PCNA will disengage its interaction with MTH2 and trigger its degradation via the proteasome in response to UV-inflicted DNA damage, although acetylation can also increase its binding with DNA polymerases β and δ^56–58^. In addition, Skp2 not only acts as a E3 ubiquitin ligase activity to promote MYC poly-ubiquitination and degradation, but also acts as a transcriptional coactivator to enhance the transcriptional activity of MYC^59,60^. Proteasome-mediated degradation is required for the transactivation of ERα and androgen receptor^61,62^. Besides, the stability and transcriptional activity of ERα are influenced by AIB1^63^. We speculated that the first event following the acetylation of ELF5 is the accumulation of ELF5 in the nucleus or its conformational changes through formation of dimers or trimers, which could increase its binding affinity for the GGA[AT] motif in *CCND1* promoter region. Moreover, acetylated ELF5 would facilitate the recruitment of co-factors such as histone acetyltransferase or histone methyltransferase, effectively increasing the transcriptional activity of ELF5 by altering the structure of chromatin. Meanwhile, ubiquitin ligases and components of the proteasome regulatory subunit would be recruited onto the *CCND1* promoter, leading to the polyubiquitination of ELF5, which is subsequently translocated from the nucleus to the cytoplasm for protein degradation. The conformational changes of ELF5 induced by acetylation would cause ELF5 to be identified as a misfolded protein, allowing it to be targeted for degradation as previously shown for fulvestrant and estrogen receptor^64^. The specific mechanism by which acetylation affects the degradation of ELF5 and its transcription activity is still unknown, but it is of great significance and deserved further research.

In conclusion, acetylation of ELF5 at lysine residues might regulate its antiproliferative activity against breast cancer. It is conceivable that acetylation-dependent repression of Cyclin D1 expression might further contribute to the anti-proliferation activity of ELF5 in breast cancer. Furthermore, the dynamic regulation of ELF5 acetylation would strengthen the function of ELF5 by targeting p300 and SIRT6. It may be possible to utilize acetyl-transferase activators or sirtuin inhibitors to promote the function of ELF5 in a disease setting, or to provide a potential target for breast cancer therapy.

## Methods

### Plasmids

Flag-Myc-ELF5 (isoform 2) were obtained from Origene. HA-tagged ELF5, GFP-tagged ELF5 and GST-tagged ELF5 were constructed by PCR and then cloned into the vector pcDNA3.1-3 × HA, pEGFP-C1, or pGEX-4T3 at the *Eco*RΙ and *Bma*HI sites. HA-p300, HA-PCAF, HA-Tip60, HA-GCN5, Flag-tagged Sirtuins (including SIRT1, SIRT2, SIRT3, SIRT4, SIRT5 and SIRT7) were kindly provided from Dr. Daming Gao (Institute of Biochemistry and Cell Biology, Shanghai). Flag-SIRT6 was obtained from Addgene (#13817). The sequence of CCND1-luc contains the promoter region −960 to +136 and was subcloned into pGL3-Basic vector (Promega, Madison, WI). HA-tagged Cyclin D1 was constructed by PCR and then cloned into the vector pCMV-HA at the *Eco*RΙ and KpnI sites.

### Cell culture and transfection

MCF7, T47D, HEK293T, HeLa cells were purchased from China Infrastructure of Cell Line Resource and used in our previous studies^65^. MCF7 cells were cultured in Eagle’s Minimum Essential Medium (EMEM) supplemented with 10% (vol/vol) fetal bovine serum (FBS) and 0.01 mg/ml human recombinant insulin. T47D cells were grown in RPMI-1640 medium supplemented with 10% (vol/vol) FBS and 0.01 mg/mL bovine insulin. HEK293T and HeLa cells were cultured in Dulbecco’s modified Eagle’s medium supplemented with 10% (vol/vol) FBS. All cell cultures were carefully maintained at 37 °C with 5% (vol/vol) CO_2_ in specific medium. Cell transfection assays were conducted as the manufacturer’s instruction of Lipofectamine 2000 (Invitrogen, Auckland, New Zealand).

### Chemicals

Garcinol (Gene Operation, Ann Arbor, MI, USA), an inhibitor of histone acetyltransferase p300 and PCAF, was added to the medium to a final concentration of 10 μM. The MG132 (a proteasomal degradation inhibitor), the C646 (a selective and competitive histone acetyltransferase p300 inhibitor) and the OSS_128167 (a selective SIRT6 inhibitor) were purchased from Selleck (Selleck, Houston, TX, USA) and used at final concentrations of 20 μM, 2 μM and 200 μM, respectively. Nicotinamide (NAM), the inhibitor of Sir2-related enzymes (Sirtuin) (Solaribo, Beijing, China) was used at a final concentration of 5 mM. The HDAC inhibitor Trichostatin A and cycloheximide (a protein synthesis inhibitor) were obtained from Sigma-Aldrich (Sigma-Aldrich, Saint Louis, Mo, USA), and used at a final concentration as follows: 2 μM, 50 μM. The CDK4/6 inhibitors palbociclib, abemaciclib, and ribociclib were obtained from MedchemExpress (MedchemExpress, New Jersey, USA) and were used at final concentrations as follows: 500 nM, 250 nM, 500 nM.

### Luciferase reporter assay

MCF7 cells and Hela cells were transfected with the appropriate plasmids for 16 h, and the medium was then changed to phenol red-free medium containing 2% charcoal-stripped fetal bovine serum (vol/vol) followed by further incubation for 24 h. The medium was replaced with complete medium and the culture was incubated for another 24 h. The relative luciferase activity of the CCND1-luc reporter was measured by dual-luciferase assay protocol (Promega, Madison, WI, USA) and expressed as fold changes.

### Western blot, immunoprecipitation, and immunofluorescence assay

Western blot (WB), immunoprecipitation (IP) and immunofluorescence assay were conducted as described previously^66^. Cells were collected and lysed with TNE lysis buffer (20 mM Tris-HCl pH 7.4, 100 mM NaCl, 1 mM EDTA, 0.5% NP-40, 10% Glycerol and complete protease inhibitor). Western blot analyses were carried out using standard protocol. In brief, samples were separated in SDS-PAGE gels, transferred to a polyvinylidene difluoride membrane (Millipore, Boston, MA, USA). The membranes were blocked in 5% milk and immunoblotted with the indicated antibodies. The bands were visualized by chemiluminescence (Advansta, San Jose, CA, USA).

For immunoprecipitations, the cells extract was incubated with the relevant antibody overnight at 4 °C, and then 60 μl Protein A/G mix magnetic beads (Bimake, Houston, USA) were added into the mixture for another 2 h at 4 °C. After washed with TNE lysis buffer for three times, the immunoprecipitated complexes were subjected to SDS-PAGE and immunoblotted with indicated antibodies.

To define the colocation of ELF5 and p300, T47D cells were cultured on glass coverslips, fixed with absolute ethyl alcohol for 20 min at 4 °C and were permeabilized with methyl alcohol for 40 min at −20 °C. Then, cells were blocked with 1% bovine serum albumin and incubated with both mouse anti-ELF5 and rabbit anti-p300 antibodies at 4 °C overnight. After washed with phosphate buffered saline three times, cells were incubated for 1 h at 4 °C with secondary antibody. The coverslips were stained with 4′,6-Diamidino-2-phenylindole (DAPI). Then the microscopic images were obtained by fluorescence microscopy.

The primary antibodies were the following: Anti-ELF5 (1:1000 for WB, 1:500 for IP, 1:50 for immunofluorescence, sc-376737, Santa Cruz Biotechnology, CA, USA), anti-ELF5 (1:1000 for WB, 1:500 for IP, sc-166653, Santa Cruz Biotechnology, CA, USA), anti-Fibrillarin (1:3000 for WB, sc-166001, Santa Cruz Biotechnology, CA, USA), anti-β-actin (1:1000 for WB, sc-47778, Santa Cruz Biotechnology, CA, USA), anti-p300 (1:3000 for WB, 1:500 for IP, 1:100 for immunofluorescence, sc-585, Santa Cruz Biotechnology, CA, USA), anti-SIRT6 (1:3000 for WB, 1:500 for IP, ab62739, Abcam, Cambridge, UK), anti-Cyclin E (1:3000 for WB, ab7959, Abcam, Cambridge, UK), anti-Flag^®^ M2 (1:2000 for WB, F1804, Sigma-Aldrich, Saint Louis, Mo, USA), anti-MYC (1:2000 for WB, PLA0001, Sigma-Aldrich, Saint Louis, Mo, USA), anti-Flag^®^ (1:2000 for WB, 1:500 for IP, F7425, Sigma-Aldrich, Saint Louis, Mo, USA), anti-GFP (1:2000 for WB, TA150052, Origene, Rockville, MD, USA), anti-Myc (1:2000 for WB, TA150121, Origene, Rockville, MD, USA), anti-GFP (1:3000 for WB, 1:500 for IP, GTX113617, GeneTex, San Antonio, TX, USA), anti-HA (1:3000 for WB, 1:500 for IP, GTX115044, GeneTex, San Antonio, TX, USA), anti-GST (1:2000 for WB, ABN116, Millipore, Boston, MA, USA), anti-acetylated-lysine (1:3000 for WB, 1:500 for IP, #9441, Cell Signaling Technology, Boston, MA, USA). Anti-Cyclin D1 (1:1000 for WB, WL01435a), anti-Cyclin B1 (1:1000 for WB, WL01760), anti-Cyclin A (1:1000 for WB, WL01841), anti-CDK1 (1:1000 for WB, WL02373), anti-CDK2 (1:1000 for WB, WL01543), anti-CDK4 (1:1000 for WB, WL02274), anti-CDK6 (1:1000 for WB, WL03460) were obtained from Wanleibio (Wanleibio, Shenyang, China).

### Xenograft model

Human breast cancer xenograft model was constructed as previously described^67^. All experiments were carried out according to the regulation set by the Ethics Committee for Biology and Medical Science of Dalian University of Technology. Female BALB/C nude mice (5–6-weeks old, 14–16 g) were obtained from Liaoning Changsheng Biotechnology and maintained under specific pathogen-free (SPF) conditions. The animals were randomly assigned to four groups and each group contained six animals. About 1 × 10^6^ MCF7 cells transfected with a control vector, ELF5-WT, ELF5-6KR, or ELF5-6KQ were suspended in 100 μl of serum-free medium, and mixed with Matrigel (BD, New Jersey, USA) at 1:1 ratio then the mixture was injected subcutaneously into the left flank of the nude mice. Ten days after the injection of the cancer cells, the size of the xenograft was determined regularly using a caliper. The tumor volume was calculated using the equation as *V* = 0.5 × (tumor length) × (tumor width)^2^. The mice were sacrificed after three weeks and the tumors were resected, photographed, and weighed. None of the experiments was allowed to exceed the tumor burden limit (10% total body weight or 2 cm in diameter).

### GST-pulldown assay

The GST pulldown assay was performed as previously described^68^. GST or GST-ELF5 fusion protein was expressed in *E. coli* BL21 (Takara, Tokyo, JAPAN) and induced with 0.1 mM Isopropyl β-D-Thiogalactoside (IPTG) at 28 °C for 6 h. Then GST or GST-ELF5 fusion protein purified with Pierce GST Spin Purification Kit (Thermo Scientific, Massachusetts, USA). The purified proteins were immobilized on the Pierce Spin Column and then incubated with HEK293T cells lysate with overexpressing p300 or SIRT6.

### In vitro acetylation and deacetylation assay

GST-ELF5 was expressed in *E. coli* BL21 and then pulled down with GST Sepharose for in vitro acetylation assay. The immunoprecipitated proteins were incubated with the lysate prepared from HEK293T cells that overexpressed HA-p300 for 2 h at 30 °C. In vitro deacetylation assay was performed with the acetylated GST-ELF5 obtained above as a substrate. Flag-SIRT6 was immunopurified with anti-Flag affinity gel (Bimake, Houston, USA) and eluted by Poly Flag Peptide (Bimake, Houston, USA). The acetylated proteins were incubated with the eluted proteins including Flag-SIRT6, and 1 mM NAD^+^ added followed by 1 h incubation at 30 °C. The proteins were resolved by SDS-PAGE and acetylated ELF5 was detected using an antibody against acetylated-lysine.

### Cell proliferation assay

Cell Counting Kit-8 assay and colony formation assays were performed according to the previous method^67^. Cells were transfected with different plasmids and then plated in 96-well plate, the cell proliferation assay was performed with Cell Counting Kit-8 (Bimake, Houston, USA). For colony formation assays, cells transfected with different plasmids were plated in 6-well plate, and then stained with crystal violet after clones were clearly visible.

### Cell cycle assay

The cells were first stained with propidium iodide and then subjected to flow cytometry to examine the cell cycle. Data were collected by CytoFLEX (Beckman) and analyzed by CytExpert software (Beckman).

### Construction of mutants and site-directed mutagenesis

ELF5 mutants (K130R, K134R, K143R, K197R, K228R, K245R), HA-p300 (S1396R, Y1397R, D1399Y), Flag-SIRT6 (H133Y), and CCND1 luciferase mutants were constructed with QuickMutation Site-Directed Mutagenesis Kit (Beyotime, Shanghai, China). These oligonucleotides and its complementary reverse oligo used as primers for mutation are listed in Supplementary Table 2. All constructs were verified by direct DNA sequencing.

### Immunohistochemical staining assay

Immunohistochemistry (IHC) staining assay was performed as previously described^66^. A total of 48 cancer tissue samples were obtained; 30 from patients from the 967rd Hospital of People’s Liberation Army and 18 from Qiqihar Medical University. The samples were incubated with a primary antibody against ELF5 (1:400, ab13581, Abcam, Cambridge, UK), SIRT6 (1:600, ab62739, Abcam, Cambridge, UK) or Cyclin D1 (1:400, WL01435a, Wanleibio, Shenyang, China). IHC staining assay was conducted according to the manufacturer’s instruction (ZSGB-BIO, Beijing, China). The IHC score of ELF5, SIRT6 and Cyclin D1 were quantified from 0 to 12 according to the percentage of stained tumor cells and staining intensity. Low expression was given a score of 0–2, moderate expression was given a score of 3–7, and high expression was allocated a score of 8–12. All individuals who donated the tissues for this study gave their consent in written form. Histologic immunohistochemical images for Figs. 5f and 7i were obtained with an Olympus IX83 Microscope Camera (Tokyo, Japan). The slides were scanned at ×20 magnification (scale bar, 500 μm).

### Gene silencing

To obtain ELF5-knockdown T47D cells, T47D cells were infected with pLKO.1 lentiviral constructs containing shRNAs against human ELF5: shELF5#1: 5′- CCG GAG CCC TGA GAT ACT ACT ATA ACT CGA GTT ATA GTA GTA TCT CAG GGC TTT TTT -3′ or shELF5#2: 5′- CCG GGG ACT GAT CTG TTC AGC AAT GCT CGA GCA TTG CTG AAC AGA TCA GTC CTT TTT -3 or’ non-target shRNA control. SiEP300, siSIRT6, siCCND1 and the negative control were purchased from GenePharma (Shanghai, China). The sequences of siRNA are listed in Supplementary Table 3. Transfection was performed with Lipofectmine 2000 (Invitrogen, Auckland, New Zealand). The knockdown efficiency was verified by western blot.

### Chromatin immunoprecipitation assay

Chromatin Immunoprecipitation (ChIP) assay was carried out as previously described^69^ and conducted with ChIP Assay Kit (Beyotime, Shanghai, China). The primers specific for the *CCND1* promoter used in the ChIP PCR analysis are listed in Supplementary Table 4.

### Anti-p300 antibody immunoprecipitated proteomics analysis

T47D cells were harvested and lysed by TNE lysis buffer, then the cell lysis was incubated with 1 μg anti-p300 antibody overnight at 4 °C. The immunoprecipitated complexes were subjected to SDS-PAGE and visualized by silver-staining. The gel was collected and washed with ultra-pure water twice. The samples were covered with destain buffer (15 mM K_3_Fe(CN)_6_, 50 mM Na_2_S_2_O_3_), then dehydrated in 50% ACN in water and 100% ACN. The dried gel was reduced with 10 mM dithiothreitol for 1 h at 55 °C and alkylated with 50 mM iodoacetamide for 30 min in darkness. The protein sample was then recovered by adding 25 mM NH_4_HCO_3_ and trypsin was added into the protein sample for digestion overnight. Next, peptide was desalted by Strata X C18 SPE column and vacuum-dried. The dried peptide was resolved in 0.1% formic acid, and analyzed by LC-MS system (EASY-nLC 1200, Thermo Scientific) coupled with Q Exactive plus. The resulting MS/MS data were used to search the Swiss-Prot database using Proteome Discoverer 2.3 (Thermo Fisher Scientific).

### Identification of acetylation site by LC-MS/MS analysis

HEK293T cells were transfected with 3 × Flag-ELF5 and p300 for 48 h, and then were lysed with TNE buffer. Flag-ELF5 protein was purified by immunoprecipitation with anti-Flag Affinity Gel (Bimake, Houston, USA) and subjected to SDS-PAGE. After SDS-PAGE and Coomassie blue staining, the appropriate bands containing Flag-ELF5 proteins were excised from the gel and collected. Samples were washed three times with 50 mM NH_4_HCO_3_ in 30% acetonitrile for 20 minutes. Then the samples were incubated with 300 μL acetonitrile for 10 min. After acetonitrile removed, the gels were reduced by incubation with 20 mM dithiothreitol at 56 °C for 30 min. Then, the gels were alkylated by 100 mM iodoacetamide in the dark for 20 min. The dried gels were digested with trypsin overnight and then the protein digest was extracted with 85% acetonitrile and 0.1% trifluoroacetic acid for twice. The remained solution was re-dissolved in 30 μL of 0.1% formic acid and analyzed by LC-MS system (EASY-nLC 1200, Thermo Scientific) coupled to an ion trap spectrometer (LTQ Velos Pro, Thermo Scientific). The resulting MS/MS data were used to search the Swiss-Prot database using Proteome Discoverer (Thermo Fisher Scientific) with MASCOT search engine software (Matrix Science).

### Statistical analysis

All blots were derived from the same experiment and were processed in parallel. Data were presented as mean ± SDs and Student’s *t* -test (unpaired, two-tailed) was used to compare two groups of independent samples. All the experiments were repeated at least three times. Statistical significance was considered at the *p* < 0.05 level.

### Ethics approval

All animal experiments and the immunohistochemical analysis using human tissues were approved by the Ethics Committee for Biology and Medical Science of Dalian University of Technology. All individuals who donated the tissues for this study gave their consent in written form.

### Reporting summary

Further information on research design is available in the Nature Research Reporting Summary linked to this article.

## Supplementary information

Supplementary Information

Reporting Summary

## Data Availability

The mass-spectrometry based proteomics data (p300-interacting proteins in human breast cancer cells) generated during this study, are publicly available in the PRIDE repository: https://identifiers.org/pride.project:PXD023240^70^. All other datasets generated and analyzed during this study (luciferase reporter assay data, immunoprecipitation and immunofluorescence assay data, xenograft model data, GST-pulldown assay data, in vitro acetylation and deacetylation assay data, chromatin Immunoprecipitation assay data, cell proliferation assay data, cell cycle assay data and immunohistochemistry data) will be made available on reasonable request from the corresponding author Dr. Huijian Wu, email address: wuhj@dlut.edu.cn. Uncropped Western blots are part of the supplementary files. The data generated and analyzed during this study are described in the following metadata record: 10.6084/m9.figshare.13522265^71^.

## References

[1] Harbeck N (2017). Breast cancer. Lancet.

[2] Siegel RL (2020). Cancer statistics, 2020. CA Cancer J. Clin..

[3] Ghoncheh M (2016). Epidemiology, incidence and mortality of breast cancer in Asia. Asian Pac. J. Cancer Prev..

[4] Swahn H (2019). Coordinate regulation of ELF5 and EHF at the chr11p13 CF modifier region. J. Cell Mol. Med..

[5] Piggin CL (2016). ELF5 isoform expression is tissue-specific and significantly altered in cancer. Breast Cancer Res..

[6] Lee HJ (2012). Elf5, hormones and cell fate.. Trends Endocrinol. Metab..

[7] Kalyuga M (2012). ELF5 suppresses estrogen sensitivity and underpins the acquisition of antiestrogen resistance in luminal breast cancer. Plos Biol..

[8] Piggin CL (2020). ELF5 modulates the estrogen receptor cistrome in breast cancer. PloS Genet..

[9] Chakrabarti R (2012). Elf5 inhibits the epithelial-mesenchymal transition in mammary gland development and breast cancer metastasis by transcriptionally repressing Snail2. Nat. Cell Biol..

[10] Yan H (2017). ELF5 in epithelial ovarian carcinoma tissues and biological behavior in ovarian carcinoma cells. Oncol. Rep..

[11] Zhang X (2019). Overexpression of E74-like factor 5 (ELF5) inhibits migration and invasion of ovarian cancer cells. Med. Sci. Monit..

[12] Li K (2017). ELF5-mediated AR activation regulates prostate cancer progression. Sci. Rep..

[13] Koyama K (2015). E74-like factor inhibition induces reacquisition of hormone sensitiveness decreasing period circadian protein homolog 1 expression in prostate cancer cells. Prostate Int..

[14] Yao B (2015). Elf5 inhibits TGF-beta-driven epithelial-mesenchymal transition in prostate cancer by repressing SMAD3 activation. Prostate.

[15] Gallego-Ortega D (2015). ELF5 drives lung metastasis in luminal breast cancer through recruitment of Gr1+ CD11b+ myeloid-derived suppressor cells. PLoS Biol..

[16] Narita T (2019). Functions and mechanisms of non-histone protein acetylation. Nat. Rev. Mol. Cell Biol..

[17] Chen L (2018). Pan-cancer analysis reveals the functional importance of protein lysine modification in cancer development. Front. Genet..

[18] Ali I (2018). Lysine acetylation goes global: from epigenetics to metabolism and therapeutics. Chem. Rev..

[19] Inuzuka H (2012). Acetylation-dependent regulation of Skp2 function. Cell.

[20] Zhang P (2014). Tumor suppressor p53 cooperates with SIRT6 to regulate gluconeogenesis by promoting FoxO1 nuclear exclusion. Proc. Natl Acad. Sci. USA..

[21] Tapias A (2017). Lysine acetylation and deacetylation in brain development and neuropathies. Genom. Proteom. Bioinform..

[22] Hsu FF (2017). Acetylation is essential for nuclear heme oxygenase-1-enhanced tumor growth and invasiveness. Oncogene.

[23] Song X (2018). Acetylation of ACAP4 regulates CCL18-elicited breast cancer cell migration and invasion. J. Mol. Cell Biol..

[24] Wang Y (2020). The role of acetylation sites in the regulation of p53 activity. Mol. Biol. Rep..

[25] Zhang J (2015). The regulation of radiosensitivity by p53 and its acetylation. Cancer Lett..

[26] Wan J (2015). PCAF-primed EZH2 acetylation regulates its stability and promotes lung adenocarcinoma progression. Nucleic Acids Res..

[27] Wang Z (2019). Acetylation of PHF5A modulates stress responses and colorectal carcinogenesis through alternative splicing-mediated upregulation of KDM3A. Mol. Cell.

[28] Dong Z (2013). Acetylation of Ets-1 is the key to chromatin remodeling for miR-192 expression. Sci. Signal..

[29] Zhang Y (2018). CREPT facilitates colorectal cancer growth through inducing Wnt/beta-catenin pathway by enhancing p300-mediated beta-catenin acetylation. Oncogene.

[30] Ni J (2014). P300-dependent STAT3 acetylation is necessary for angiotensin II-induced pro-fibrotic responses in renal tubular epithelial cells. Acta Pharm. Sin..

[31] Qiu Y (2006). HDAC1 acetylation is linked to progressive modulation of steroid receptor-induced gene transcription. Mol. Cell.

[32] Han Z (2017). Profiling cellular substrates of lysine acetyltransferases GCN5 and p300 with orthogonal labeling and click chemistry. ACS Chem. Biol..

[33] Ross NT (2020). CPSF3-dependent pre-mRNA processing as a druggable node in AML and Ewing’s sarcoma. Nat. Chem. Biol..

[34] Lee HJ (2011). Lineage specific methylation of the Elf5 promoter in mammary epithelial cells. Stem Cells.

[35] Dancy BM (2015). Protein lysine acetylation by p300/CBP. Chem. Rev..

[36] Wang Y (2019). p300 acetyltransferase is a cytoplasm-to-nucleus shuttle for SMAD2/3 and TAZ nuclear transport in transforming growth factor beta-stimulated hepatic stellate cells. Hepatology.

[37] Gil J (2017). Lysine acetylation and cancer: a proteomics perspective. J. Proteom..

[38] Carafa V (2019). Dual tumor suppressor and tumor promoter action of sirtuins in determining malignant phenotype. Front. Pharm..

[39] Bosch-Presegue L (2011). The dual role of sirtuins in cancer. Genes Cancer.

[40] Liu L (2012). Prevention of ERK activation involves melatonin-induced G(1) and G(2) /M phase arrest in the human osteoblastic cell line hFOB 1.19. J. Pineal. Res..

[41] Liu Q (2008). miR-16 family induces cell cycle arrest by regulating multiple cell cycle genes. Nucleic Acids Res..

[42] Hermosilla EV (2018). SALL2 represses cyclins D1 and E1 expression and restrains G1/S cell cycle transition and cancer-related phenotypes. Mol. Oncol..

[43] Ferguson KL (2000). The Rb-CDK4/6 signaling pathway is critical in neural precursor cell cycle regulation. J. Biol. Chem..

[44] Morgan, D.O., The cell cycle: principles of control. (New Science Press, 2007).

[45] Dulic V (1992). Association of human cyclin E with a periodic G1-S phase protein kinase. Science.

[46] Albanese C (1995). Transforming p21ras mutants and c-Ets-2 activate the cyclin D1 promoter through distinguishable regions. J. Biol. Chem..

[47] Tyagi N (2018). ETV4 facilitates cell-cycle progression in pancreatic cells through transcriptional regulation of cyclin D1. Mol. Cancer Res..

[48] Stacey DW (2003). Cyclin D1 serves as a cell cycle regulatory switch in actively proliferating cells. Curr. Opin. Cell Biol..

[49] Wang D (2016). Acetylation-regulated interaction between p53 and SET reveals a widespread regulatory mode. Nature.

[50] Kim MY (2006). Acetylation of estrogen receptor alpha by p300 at lysines 266 and 268 enhances the deoxyribonucleic acid binding and transactivation activities of the receptor. Mol. Endocrinol..

[51] Escamilla-Hernandez R (2010). Genome-wide search identifies Ccnd2 as a direct transcriptional target of Elf5 in mouse mammary gland. BMC Mol. Biol..

[52] Gronroos E (2002). Control of Smad7 stability by competition between acetylation and ubiquitination. Mol. Cell.

[53] Wang X (2004). Inhibition of p53 degradation by Mdm2 acetylation. FEBS Lett..

[54] Du Z (2010). DNMT1 stability is regulated by proteins coordinating deubiquitination and acetylation-driven ubiquitination. Sci. Signal.

[55] Jiang W (2011). Acetylation regulates gluconeogenesis by promoting PEPCK1 degradation via recruiting the UBR5 ubiquitin ligase. Mol. Cell.

[56] Yu Y (2009). Proliferating cell nuclear antigen is protected from degradation by forming a complex with MutT Homolog2. J. Biol. Chem..

[57] Cazzalini O (2014). CBP and p300 acetylate PCNA to link its degradation with nucleotide excision repair synthesis. Nucleic Acids Res.

[58] Naryzhny SN (2004). The post-translational modifications of proliferating cell nuclear antigen: acetylation, not phosphorylation, plays an important role in the regulation of its function. J. Biol. Chem..

[59] Kim SY (2003). Skp2 regulates Myc protein stability and activity. Mol. Cell.

[60] von der Lehr N (2003). The F-box protein Skp2 participates in c-Myc proteosomal degradation and acts as a cofactor for c-Myc-regulated transcription. Mol. Cell.

[61] Reid G (2003). Cyclic, proteasome-mediated turnover of unliganded and liganded ERalpha on responsive promoters is an integral feature of estrogen signaling. Mol. Cell.

[62] Kang Z (2002). Involvement of proteasome in the dynamic assembly of the androgen receptor transcription complex. J. Biol. Chem..

[63] Shao W (2004). Coactivator AIB1 links estrogen receptor transcriptional activity and stability. Proc. Natl Acad. Sci. USA..

[64] Pike AC (2001). Structural insights into the mode of action of a pure antiestrogen. Structure.

[65] Wang J (2019). Circadian protein BMAL1 promotes breast cancer cell invasion and metastasis by up-regulating matrix metalloproteinase9 expression. Cancer Cell Int..

[66] Bai XY (2018). Kruppel-like factor 9 down-regulates matrix metalloproteinase 9 transcription and suppresses human breast cancer invasion. Cancer Lett..

[67] Xu Z (2018). Checkpoint suppressor 1 suppresses transcriptional activity of ERalpha and breast cancer cell proliferation via deacetylase SIRT1. Cell Death Dis..

[68] Liu Y (2015). FOXK2 transcription factor suppresses ERalpha-positive breast cancer cell growth through down-regulating the stability of ERalpha via mechanism involving BRCA1/BARD1. Sci. Rep..

[69] Jia Z (2018). U-box ubiquitin ligase PPIL2 suppresses breast cancer invasion and metastasis by altering cell morphology and promoting SNAI1 ubiquitination and degradation. Cell Death Dis..

[70] Li, X. et al. human breast cancer cell, LC-MSMS. PRIDE Archive https://identifiers.org/pride.project:PXD023240 (2021).

[71] Li, X. et al. Metadata supporting the article: acetylation of ELF5 suppresses breast cancer progression by promoting its degradation and targeting CCND1. figshare 10.6084/m9.figshare.13522265 (2021). 10.1038/s41698-021-00158-3 PMC7979705 33742100

