# Supplementary material for: Acetylation of ELF5 suppresses breast cancer progression by promoting its degradation and targeting CCND1
Source: NPJ Precis Oncol. 2021 Mar 19;5:20. doi: 10.1038/s41698-021-00158-3 (PMC7979705; doi:10.1038/s41698-021-00158-3)

Supplementary information

Acetylation of ELF5 suppresses breast cancer progression by promoting its degradation and targeting *CCND1*

Xiahui Li[1], Shujing Li[1], Bowen Li[1], Yanan Li[1], Aman Sattout [1], Kangkai Xia[1], Yuxi Yang[1], Bashir Ahmad[1], Huijian Wu[1]*

1. School of Bioengineering & Key Laboratory of Protein Modification and Disease, Liaoning Province, Dalian University of Technology, Dalian 116024, China.

*Correspondence: wuhj@dlut.edu.cn

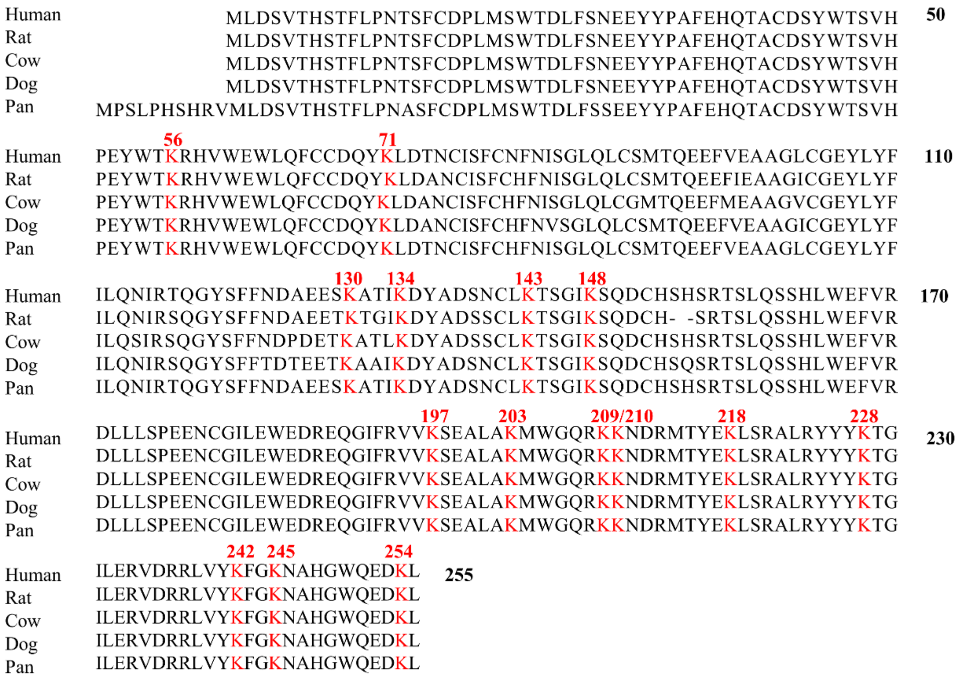

Supplementary Figure 1**.** Alignment of ELF5 sequences from different species. Alignment of five different ELF5 vertebrate orthologs with the conserved lysine residues numbered and colored in red.

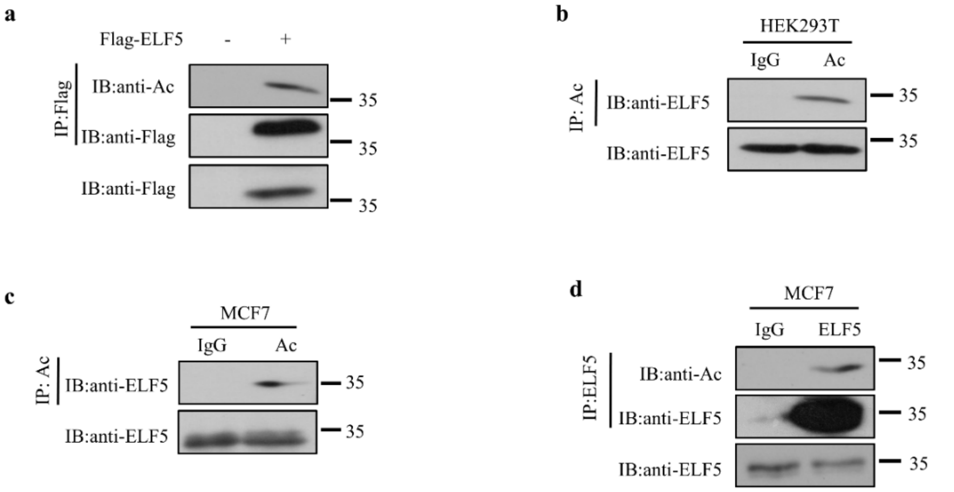

Supplementary Figure 2. Acetylation of ELF5 in HEK293T and MCF7 cells.
(a) Acetylation of exogenous ELF5. HEK293T cells were transfected with Flag-ELF5, immunoprecipitated with anti-Flag affinity gel and immunoblotted with anti-Ac Ab. (b) Acetylation of endogenous ELF5 in HEK293T cells. Cell lysis was subjected to immunoprecipitation with anti-AcK Ab or normal IgG, followed by western blot with anti-ELF5 Ab. (c-d) Acetylation of endogenous ELF5 in MCF7 cells. Cell lysis was subjected to immunoprecipitation with anti-AcK Ab or normal IgG, followed by western blot with anti-ELF5 Ab (c). Cell lysis was subjected to immunoprecipitation with anti-ELF5 Ab or normal IgG, followed by western blot with anti-AcK Ab (d).

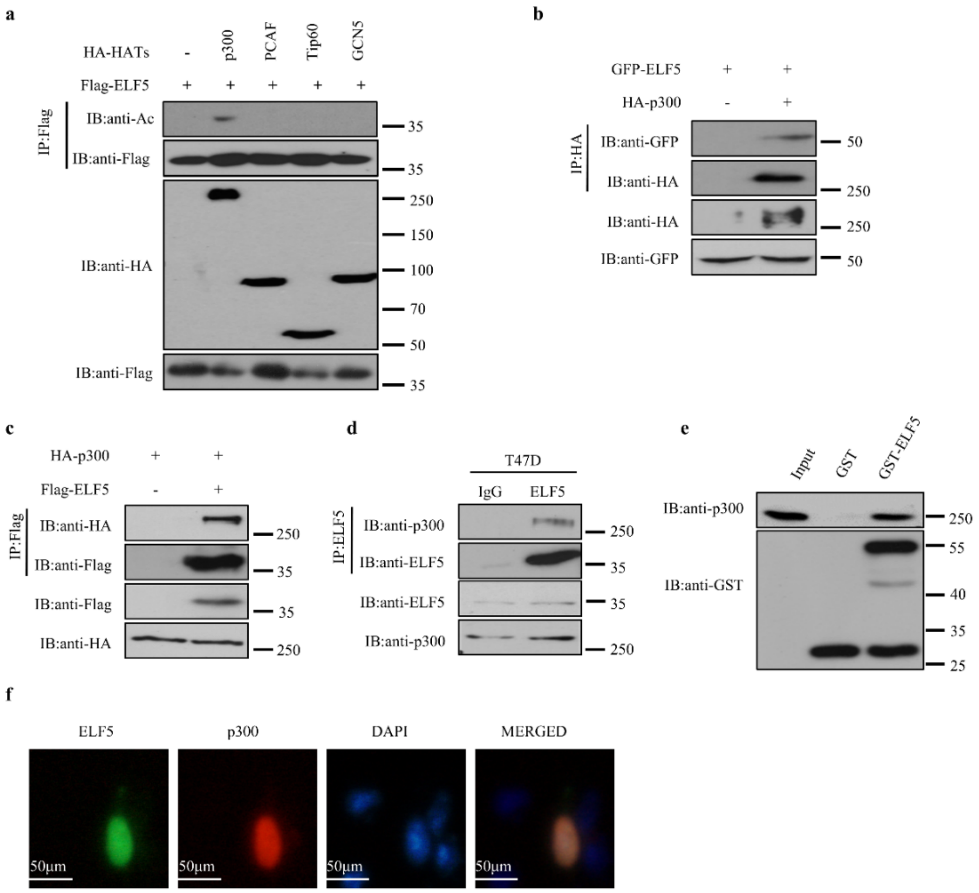

Supplementary Figure 3. ELF5 interacts with p300 both *in vitro* and *in vivo*.

(a) p300-mediated acetylation of exogenous ELF5. Hela cells were co-transfected with Flag-ELF5 and different HA-tagged HATs, HA-p300, HA-PCAF, HA-GCN5, or HA-Tip60, and the cell lysates were immunoprecipitated with anti-Flag affinity gel and immunoblotted with the anti-AcK Ab. (b) Exogenous ELF5 interacted with p300 *in vivo*. HEK293T cells expressing HA-p300 only or HA-p300 and GFP-ELF5 were prepared and subjected to affinity purification with anti-HA Ab, followed by Western blot with the indicated antibodies. (c) Exogenous ELF5 interacted with p300 *in vivo*. HEK293T cells transfected with Flag-ELF5 only, or with Flag-ELF5 and HA-p300 were subjected to co-immunoprecipitation with anti-Flag affinity gel followed by immunoblotting with antibodies as indicated. (d) Endogenous ELF5 interacted with p300 *in vivo*. T47D cells were subjected to immunoprecipitation with anti-ELF5 antibody or normal IgG followed by Western blot with the indicated antibodies. (e) ELF5 interacted with p300 *in vitro*. GST pull-down assays showing the interaction between GST-fusion ELF5 and endogenous p300 in T47D cells. (f) ELF5 and p300 were colocalized in the nucleus. Immunofluorescence showing the localization of ELF5 (blue) and p300(red) in the nucleus (blue) of T47D cells.

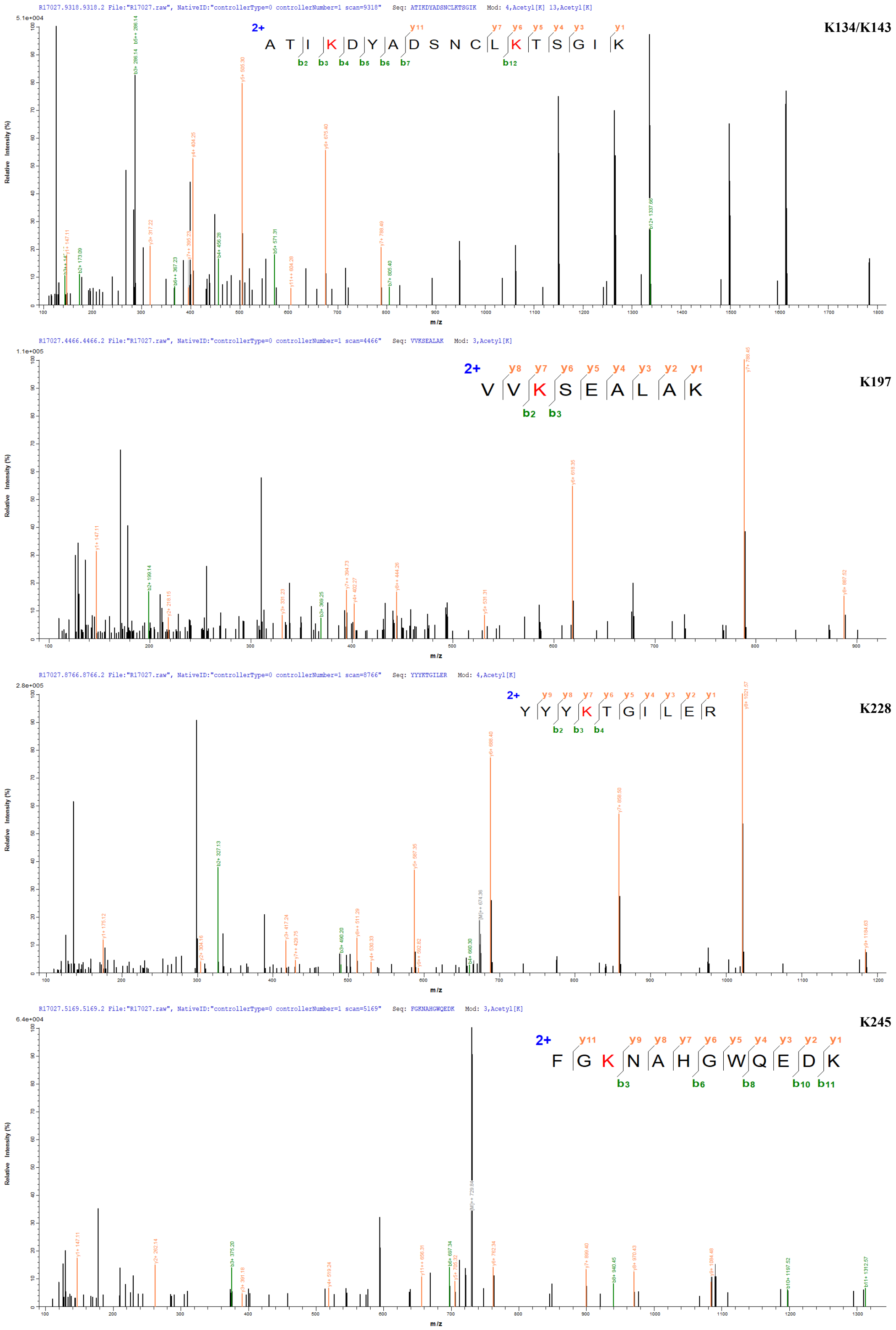

Supplementary Figure 4. Mass spectrometry analysis of the ELF5-derived peptides containing acetylated K134, K143, K197, K228, and K245.

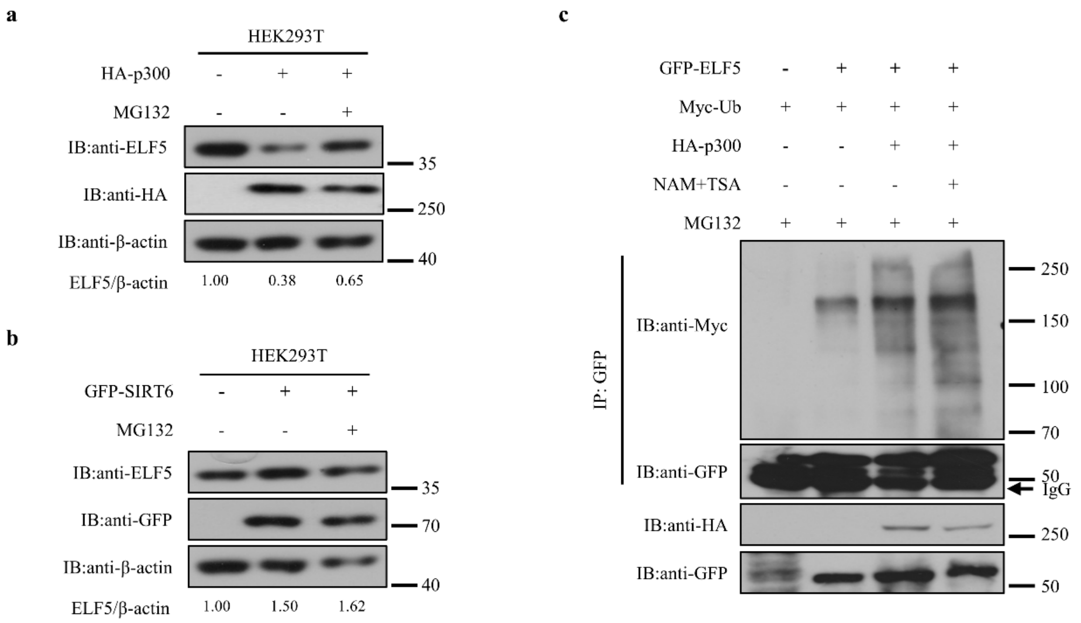

Supplementary Figure 5. Acetylation promotes the ubiquitin-proteasome degradation of ELF5.

(a) The protein level of ELF5 influenced by p300. HEK239T cells overexpressing HA-p300 in the absence or presence of 20 μM MG132 were prepared and subjected to western blot with the indicated antibodies. (b) The protein level of ELF5 influenced by SIRT6. HEK293T cells overexpressing GFP-SIRT6 in the absence or presence of 20 μM MG132 were prepared and subjected to western blot with the indicated antibodies. (c) The ubiquitination level of ELF5 influenced by p300. Ubiquitination levels of GFP-ELF5 in HEK293T cells without or with overexpression with HA-p300. The cells were treated without or with NAM plus TSA before being harvested.

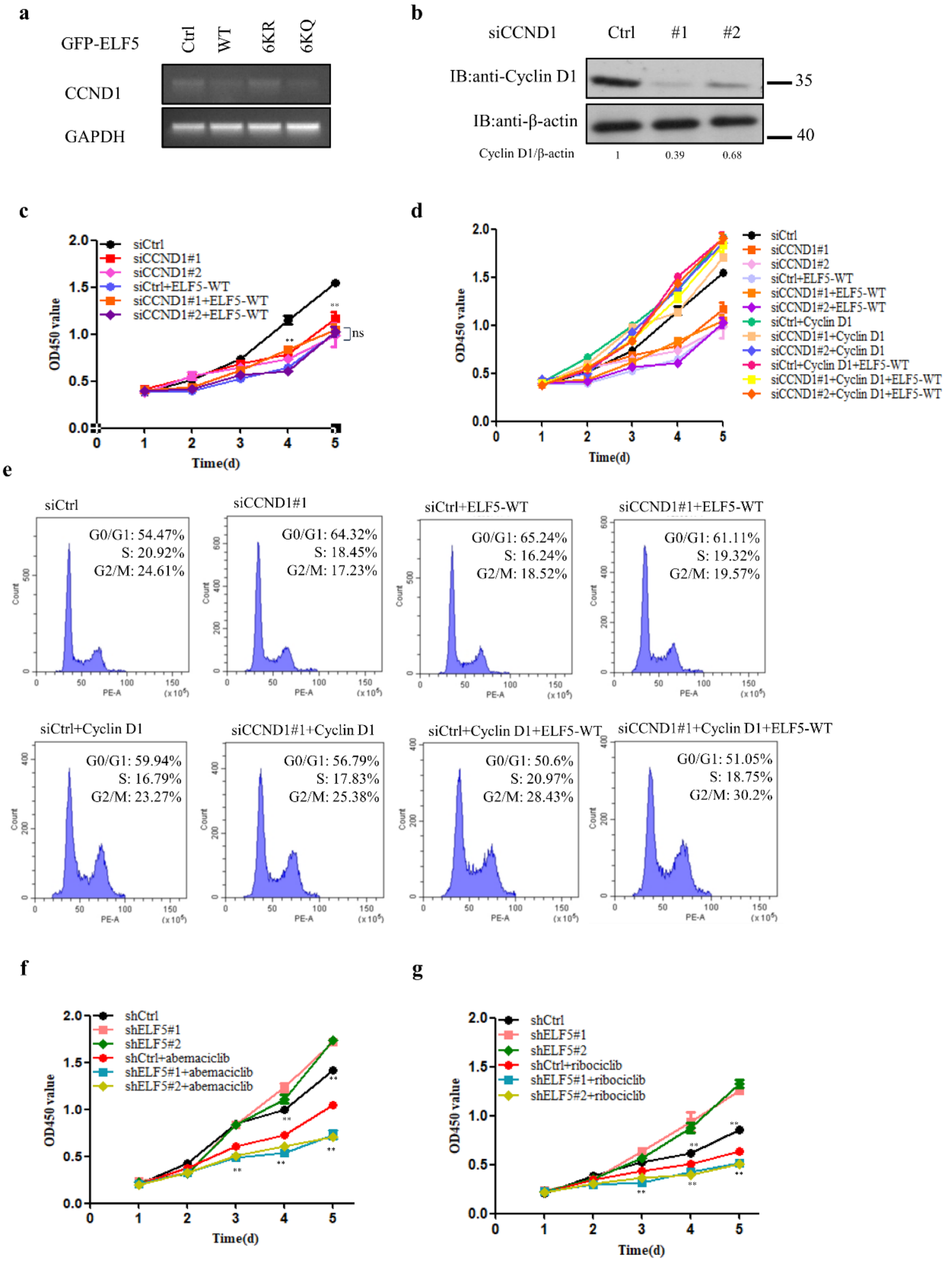

Supplementary Figure 6. ELF5 inhibit cell proliferation through CCND1.

(a) Cells that overexpressed with GFP-ELF5 WT, GFP-ELF5 6KR, GFP-ELF5-6KQ or the control vector were analyzed for CCND1 mRNA levels. (b) Western-blot detection of Cyclin D1 expression in MCF7 cells expressing a siCtrl or siCCND1. (c) Growth curves of MCF7 cells that stably overexpressed with GFP-ELF5 without or with siCCND1 as measured by CCK8 assay. Data are the means ± SDs from three determinations. **p*<0.05; ***p*<0.01. (d) Growth curves of MCF7 cells performed a CCND1 rescue experiment overexpressing ELF5 wildtype. Data are the means ± SDs from three determinations. **p*<0.05; ***p*<0.01. (e) Flow cytometry analysis of the cell

cycle showing the distribution of MCF7 cells stably transfected with ELF5 WT with a Cyclin D1 rescue. (f-g) Growth curves of T47D cells expressing a shCtrl or shELF5 without or with abemaciclib (e) or ribociclib (f) treatment as measured by CCK8 assay. *p<0.05; **p<0.01; ns, no significance.

Supplementary Table 1. List of identified p300-interacting proteins

AAMP	ABCE1	ABCF1	ABCF2	ABCF3	ACACA	ACAD9	ACLY
ACSL3	ACTB	ADAR	ADRM1	AGK	AGPS	AICDA	AIFM1
AIMP1	AIMP2	AKAP8	ALDH18A1	ALDOA	ALYREF	AMOT	AP1B1
AP1G1	AP1M1	AP2A1	AP2A2	AP2B1	AP2M1	AP3B1	ARCN1
ARF5	ARMC6	ASIC1	ASNS	ATG3	AUP1	BAG6	BAZ1B
BCCIP	BSN	BTAF1	BUB3	BUD23	BZW1	BZW2	C1QBP
C2CD5	CALR	CALU	CAND1	CASC3	CBL	CCAR2	CCDC6
CCDC9	CCT6A	CDC16	CDC27	CDC40	CDC5L	CDC73	CDK1
CDK12	CDK9	CENPU	CFDP1	CHD3	CDERP	CDMP6	CHTF18
CHTOP	CKAP4	CLINT1	CLNS1A	CLPB	CLPX	CLU	CMC1
CMC2	CMTR1	CNN3	COPA	COPB1	COPB2	COPE	COPG1
COPS4	COPS5	COPS6	CPSF1	CPSF3	CPSF6	CPSF7	CSDE1
CSE1L	CSN2	CSTF3	CTR9	CTU2	CUL1	CUL3	CUL4B
CWF19L1	DAXX	DBR1	DCD	DCTN2	DDB1	DDOST	DDX1
DDX17	DDX18	DDX21	DDX23	DDX27	DDX3X	DDX41	DDX42
DDX46	DDX47	DDX5	DDX50	DDX54	DDX55	DDX56	DDX6
DHCR7	DHX15	DHX16	DHX30	DHX36	DHX38	DHX57	DHX9
DIS3	DKC1	DNM2	DNMT1	DNPEP	DOCK7	DPM1	DRG1
DRG2	DUS3L	EBNA1BP2	ECPAS	EDC3	EEA1	EEF1B2	EEF1D
EEF1G	EEF2	EHD4	EIF2A	EIF3A	EIF3B	EIF3C	EIF3D
EIF3E	EIF3F	EIF3G	EIF3H	EIF3I	EIF3J	EIF3L	EIF3M
ELAVL1	ELF5	ELP1	ELP2	ELP3	EMC1	EMD	ENAH
ERI1	ESYT2	EWSR1	EXOC3	FAF1	FAF2	FAS	FBLL1
FHL1	FIP1L1	FKBP8	FLII	FLNA	FOXK1	FOXK2	FRAS1
FRG1	FUBP3	FUS	FXR1	FYCO1	FZD6	G3BP1	GNL1
G3BP2	GANAB	GCFC2	GCN1	GFPT1	GFPT2	GNE	GTPBP4
GNL3	GPHN	GPS1	GRN	GRSF1	GRWD1	GTF2I	HDGF
GYS1	HACD3	HADHA	HADHB	HAT1	HBS1L	HDAC1	HLA-A
HDHD5	HELLS	HES6	HGH1	HIC2	HK1	HK2	HNRNPM
HMGB1	HMMR	HNRNPC	HNRNPD	HNRNPF	HNRNPK	HNRNPL	IFIT1
HNRNPR	HNRNPU	HSPA5	HSPA9	IDH3A	IDH3B	IDH3G	IPO5
IFRD1	IGBP1	IGHG1	IK	ILF2	ILF3	IPO4	KIF5B
IPO7	IPO9	ITGAL	IWS1	KHSRP	KIF11	KIF2A	KRR1
KIFC1	KLC1	KLC2	KLF16	KLF4	KPNB1	KRI1	LDHB
LAMP2	LARP1	LARP4	LARP7	LASP1	LCK	LDHA	MAP4
LEO1	LETM1	LGR6	LSG1	LUC7L	LYAR	MAGT1	MCM3
MAP7	MASP2	MATR3	MAZ	MCAT	MCCC2	MCM2	MFAP1
MCM4	MCM5	MCM6	MCM7	MCMBP	MEMO1	MEPCE	MPP6
MFN1	MIER1	MLST8	MMS19	MOGS	MORN1	MOV10	MYH10
MRM3	MRTO4	MSH2	MSH6	MTA2	MTDH	MTREX	NCBP1
MYH9	NAA15	NACA	NADK	NAMPT	NASP	NAT10	NMD3
NCBP3	NCLN	NEK9	NELFE	NEMF	NFS1	NKRF	NOP58

NMT2	NOB1	NONO	NOP14	NOP2	NOP53	NOP56	NUB1
NOP9	NOSIP	NPM1	NR1H3	NRDC	NSDHL	NSF	OSBP
NUCKS1	NUMA1	NUO93	OGDHL	OLA1	OPA1	OPA6	PALLD
OTUB1	OXSR1	PA2G4	PABPC1	PABPC4	PABPN1	PAF1	PCNA
PANK4	PARG	PARP1	PATL1	PCAT1	PCBP1	PCBP2	PELO
PDCD2	PDCD4	PDE12	PDIA3	PDIA4	PDIA5	PDIA6	PHF6
PELP1	PGAM1	PGAM5	PHAX	PHB	PHB2	PHF23	PNO1
PHGDH	PINX1	PITX2	PKN2	PLCG1	PLOD1	PLRG1	POP1
PNPT1	POLDIP3	POLR1C	POLR2A	POLR2A	POLR2L	POLR3C	PREB
PPAN	PPIL2	PPIL4	PPM1G	PPP1R3A	PRCC	PRDX4	PRPF8
PRKDC	PRMT1	PRPF19	PRPF3	PRPF31	PRPF4B	PRPF6	PSMD7
PRPS1	PSD2	PSIP1	PSMD1	PSMD2	PSMD3	PSMD6	PUM1
PSMD8	PSME3	PSPC1	PTBP1	PTBP2	PTCD3	PUF60	RACK1
PURA	PURB	PUS7	PWP1	PYCR2	PYM1	RABL6	RBM17
RALY	RAN	RBBP4	RBBP5	RBBP6	RBM14	RBM15	RBM7
RBM23	RBM25	RBM26	RBM27	RBM28	RBM39	RBM4	RFC2
RBMX	RBP2	RCC1	RCC2	RCN1	RCN2	RDH11	ROBO4
RFC3	RFC4	RGS11	RIC8A	RIF1	RIOK1	RNF40	RPN1
ROCK1	ROCK2	RPAP2	RPAP3	RPF1	RPF2	RPL14	RTN4
RPN2	RRM1	RSBN1	RSF1	RTCB	RTF1	RTF2	SBNO1
RTRAF	RWDD1	SACM1L	SAFB	SAFB2	SAFB3	SART3	SEPTIN2
SCAF4	SCNM1	SCO2	SCRIB	SCYL1	SEC62	SEC63	SF3B2
SEPTIN7	SESN2	SET	SF3A1	SF3A2	SF3A3	SF3B1	SLC3A2
SF3B3	SFPQ	SFXN1	SLBP	SLC1A5	SLC25A10	SLC25A6	SND1
SLC7A5	SLK	SLTM	SMC1A	SMC2	SMC3	SMC4	SRP68
SNRPA	SNW1	SNX1	SNX9	SP3	SPAG5	SPART	SRSF4
SRP72	SRPK1	SRPK2	SRRM2	SRRT	SRSF1	SRSF2	STAU1
SRSF5	SRSF6	SRSF7	SRSF9	SSB	SSRP1	STAT3	SUDS3
STK26	STK39	STON2	STRAP	STRBP	STT3A	STT3B	SYNM
SUPT4H1	SUPT5H	SUPT6H	SURF2	SURF6	SYK	SYNC	SHOC2
TAF15	TAF6	TBL2	TCEA1	TECR	TENT4A	TES	TNPO1
TFB1M	TFPC	TIMM44	TIMM50	TLN1	TMC5	TMEM201	TRIM28
TNPO3	TOE1	TPM4	TPX2	TRA2A	TRA2B	TRIM21	TXLNA
TRMT1	TRMT1L	TSR1	TSR3	TTC4	TTLL9	TUFM	TFAP2C
TXLNG	TXNL4A	TYW5	U2AF1	U2AF2	U2SURP	UBA5	USP9X
UBE3C	UFD1	UFL1	UMPS	UPF1	UQCRC2	USO1	UBE2S
UTP18	UTS2	VAC14	VCP	VPS35	VPS4A	VPS72	XAB2
WBP4	WDHD1	WDR11	WDR44	WDR82	WEE1	WRAP53	WBP11
XPO1	XPO5	XPOT	XRCC5	XRCC6	XRN2	YJU2	YLPM1
ZC3H4	ZC3HC1	ZFP91	ZFR	ZNF24	ZNF469	ZNF48	ZYX
ZW10							

Supplementary Table 2. The primers for Site-directed mutagenesis

	sense (5'-3')	antisense (5'-3')
K130R	gacgctgaagaaagcagggccaccatcaaaga	tctttgatggtggccctgctttcttcagcgtc
K134R	gcaaggccaccatcagagactatgctgattcc	ggaatcagcatagtctctgatggtggccttgc
K143R	gattccaactgcttgagaacaagtggcatcaaaa	ttttgatgccacttgttctcaagcagttggaatc
K197R	aatttttcgggtggttagatcggaagccctg	cagggcttccgatctaaccacccgaaaaatt
K228R	cctgagatactactatagaacaggaattttggagc	gctccaaaattcctgttctatagtagtatctcagg
K245R	tagtgtacaaatttggaagaaatgcacacgggtg	cacccgtgtgcatttcttccaaatttgtacacta
K130Q	gacgctgaagaaagccaggccaccatcaaag	ctttgatggtggcctggctttcttcagcgtc
K134Q	gcaaggccaccatccaagactatgctgattcc	ggaatcagcatagtcttggatggtggccttgc
K143Q	tgattccaactgcttgcaaacaagtggcatcaaa	tttgatgccacttgtttgcaagcagttggaatca
K197Q	gaatttttcgggtggttcaatcggaagccctgg	ccagggcttccgattgaaccacccgaaaaattc
K228Q	ccctgagatactactatcaaacaggaattttggag	ctccaaaattcctgtttgatagtagtatctcaggg
K245Q	ttagtgtacaaatttggacaaaatgcacacgggtg	cacccgtgtgcattttgtccaaatttgtacactaa
S1396R	agaggagagtatacatacgttacctcgatagtgttc	gaacactatcgaggtaacgtatgtatactctcctct
Y1397R	ggagagtatacatatctcgcctcgatagtgttcat	atgaacactatcgaggcgagatatgtatactctcc
D1399Y	gtatacatatcttacctctatagtgttcatttcttcc	ggaagaaatgaacactatagaggtaagatatgtatac
H133Y	aaactggcagagctctacgggaacatgtttgt	acaaacatgttcccgtagagctctgccagttt
Position 1-mut	aaggacaagatgaaggccatgctggccaccatc	gatggtggccagcatggccttcatcttgtcctt
Position 2-mut	tcgtggcgttcttggccatgcgcccattctgc	gcagaatgggcgcatggccaagaacgccacga

Supplementary Table 3. The siRNA sequence for RNA interference

	sense (5’-3’)	antisense (5’-3’)
siEP300#1	ugacacaggcaggcuugactt	gucaagccugccugugucatt
siEP300#2	cagagcaguccuggauuagtt	cuaauccaggacugcucuguu
siSIRT6#1	gaaugugccaaguguaagatt	ucuuacacuuggcacauucuu
siSIRT6#2	gcuacguugacgaggucautt	augaccucgucaacguagctt
siCCND1#1	ggagaacaaacagaucauctt	gaugaucuguuuguucucctc
siCCND1#2	gcacgauuucauugaacactt	gucaagccuhccugugucatt
Negative control	uucuccgaacgugucacgutt	acgugacacguucggagaatt

Supplementary Table 4. The primers for ChIP analysis

	sense (5'-3')	antisense (5'-3')
region 1	gaacacctatcgattttgc	gaccagtcggtccttgcg
region 2	aaggtaggaaggcagcccg	ccatatccaagccggcaga
region 3	ggtgtcgccgcgccccag	ggaggctccaggactttgc

Figure 1b

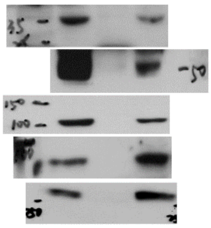

Figure 2a

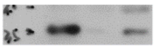

Figure 2b

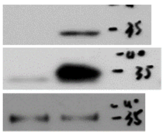

Figure 2c

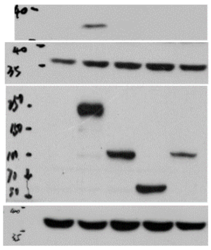

Figure 2d

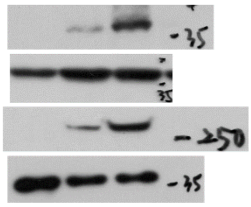

Figure 2f

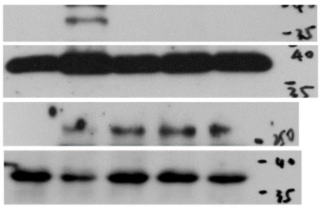

Figure 2g

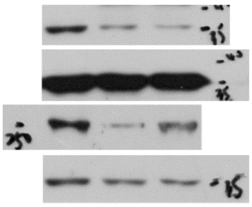

Figure 2h

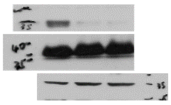

Figure 2i

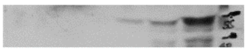

Figure 3e

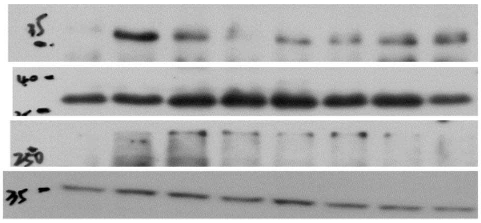

Figure 3g

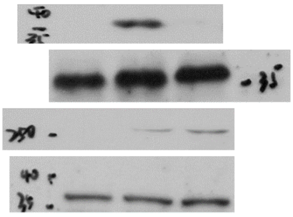

Figure 4a

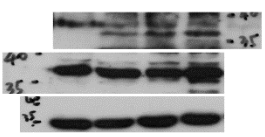

Figure 4b

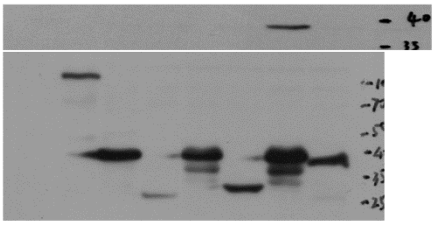

Figure 4c

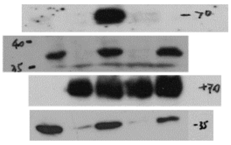

Figure 4d

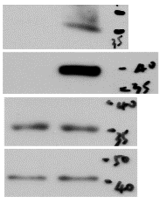

Figure 4e

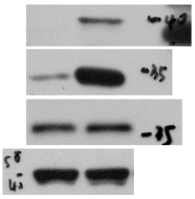

Figure 4f

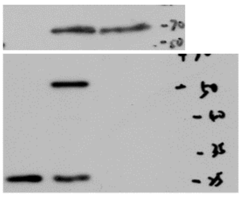

Figure 4g

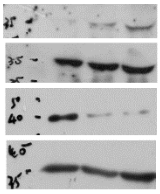

Figure 5b

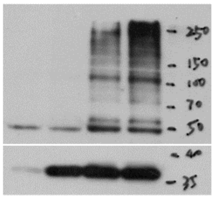

Figure 4h

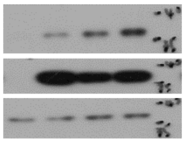

Figure 4i

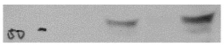

Figure 5a

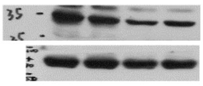

Figure 7b

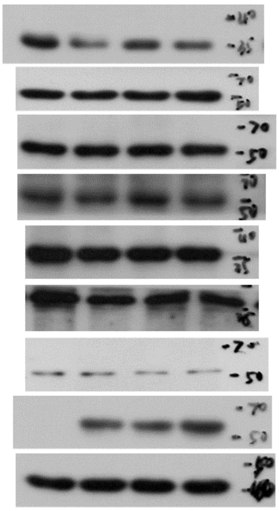

Figure 5c

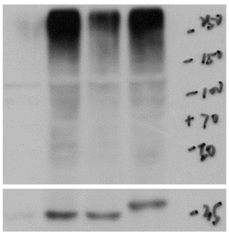

Figure 5d

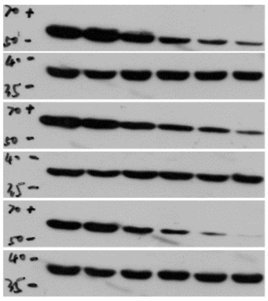

Figure 7g

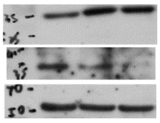

Supplementary Figure 2a

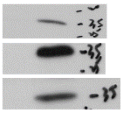

Supplementary Figure 2b

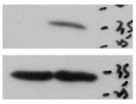

Supplementary Figure 2c

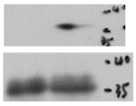

Supplementary Figure 2d

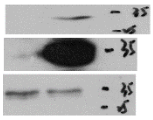

Supplementary Figure 3a

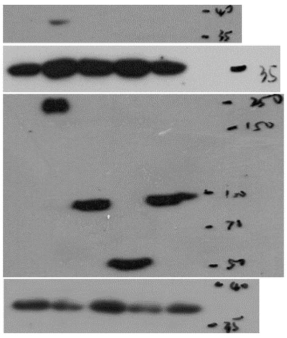

Supplementary Figure 3b

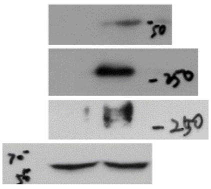

Supplementary Figure 3c

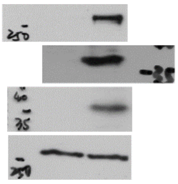

Supplementary Figure 3d

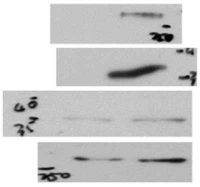

Supplementary Figure 5c

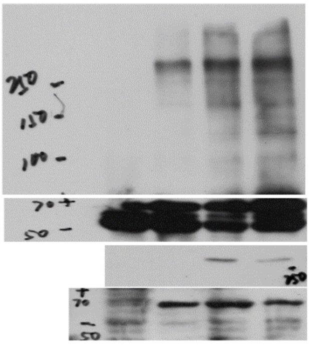

Supplementary Figure 3e

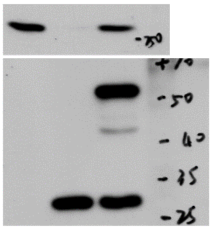

Supplementary Figure 5a

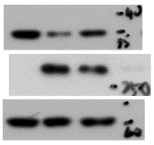

Supplementary Figure 5b

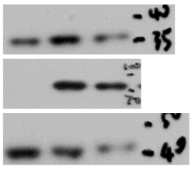

Supplementary Figure 6b

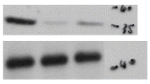

Figure 1b

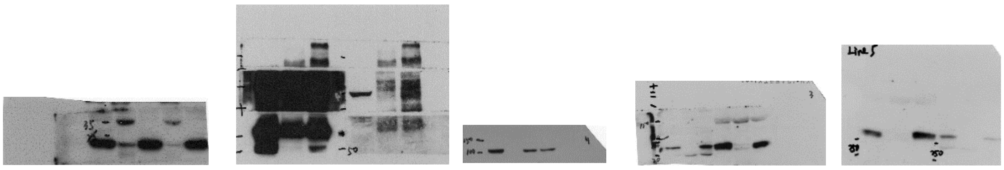

Figure 2a

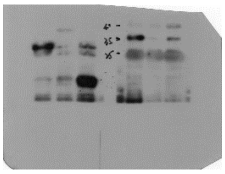

Figure 2b

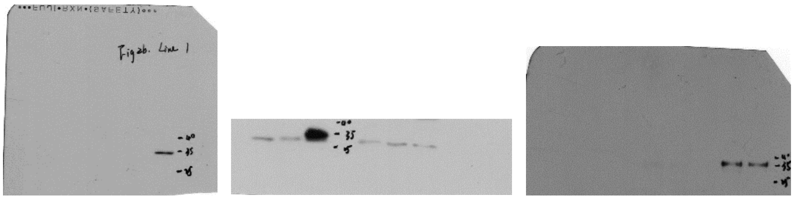

Figure 2c

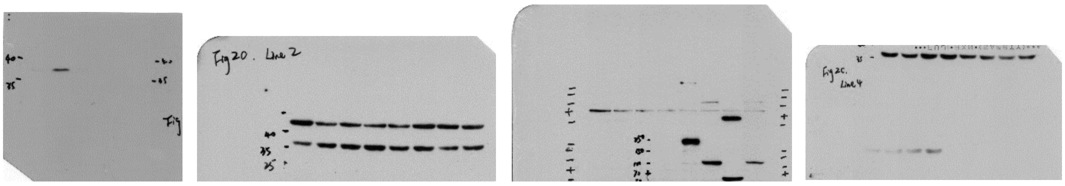

Figure 2d

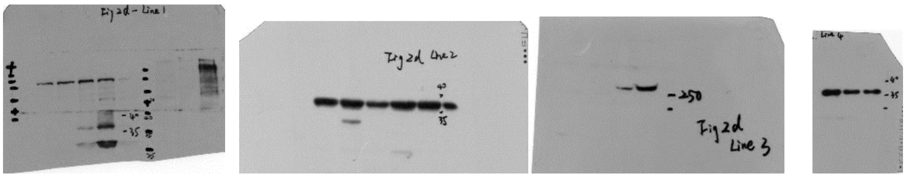

Figure 2f

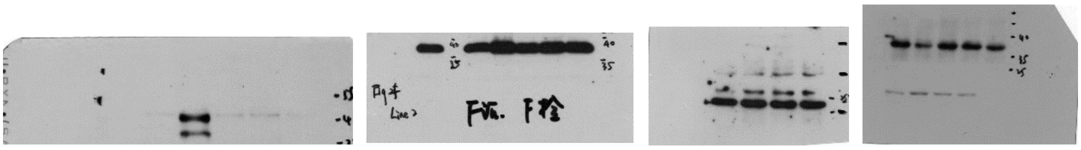

Figure 2g

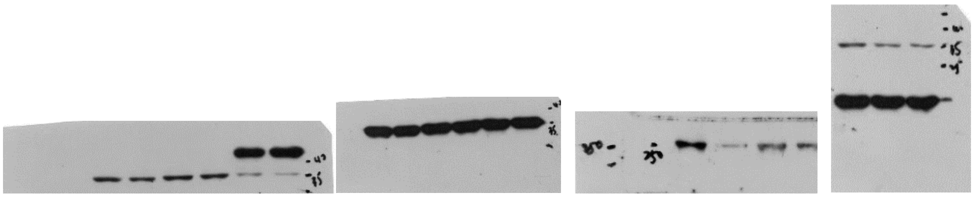

Figure 2h

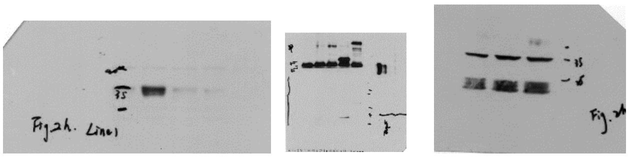

Figure 2i

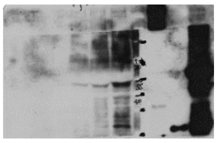

Figure 3e

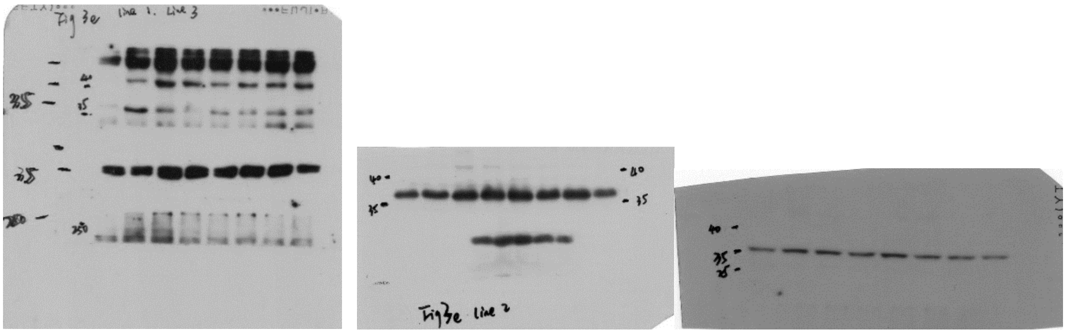

Figure 3g

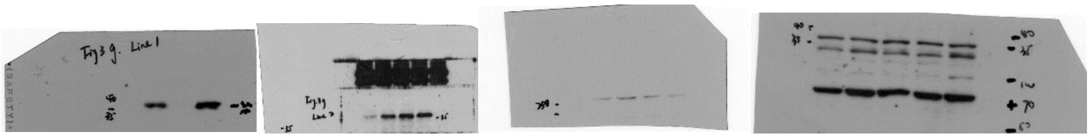

Figure 4a

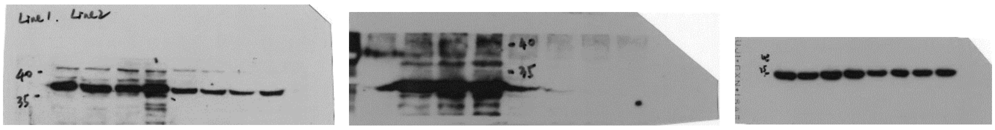

Figure 4b

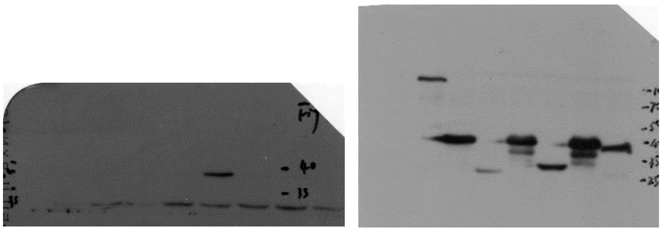

Figure 4c

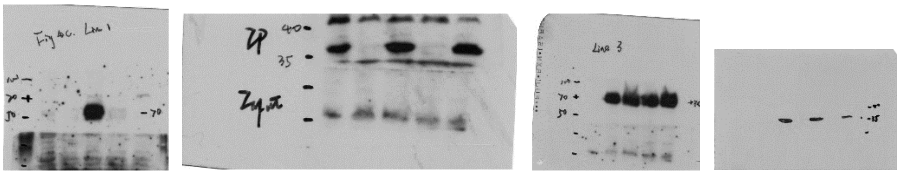

Figure 4d

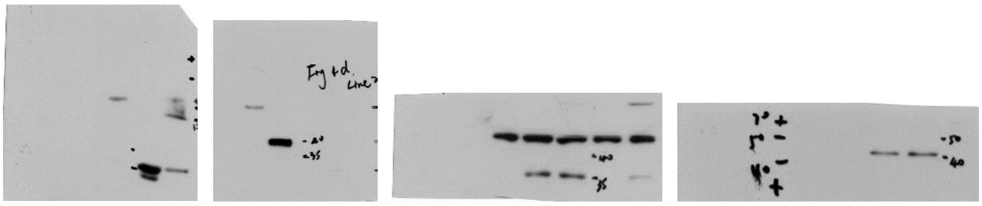

Figure 4e

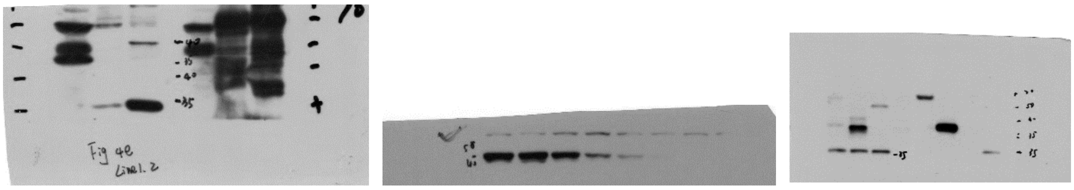

Figure 4f

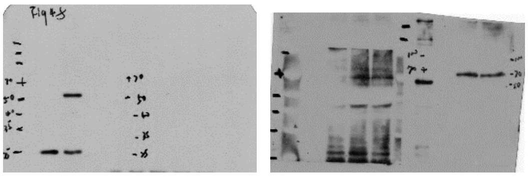

Figure 4g

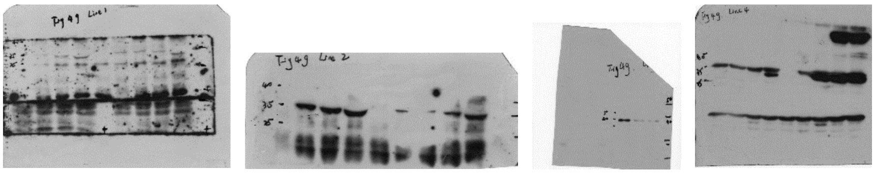

Figure 4h

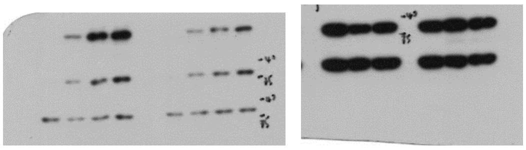

Figure 4i

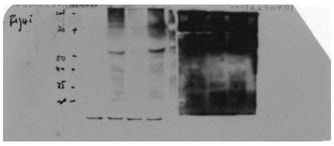

Figure 5a

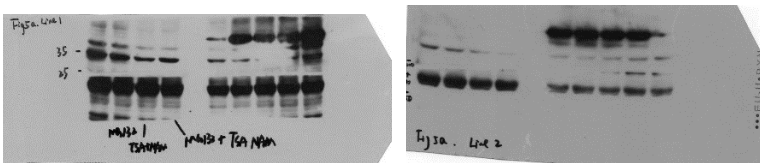

Figure 5b

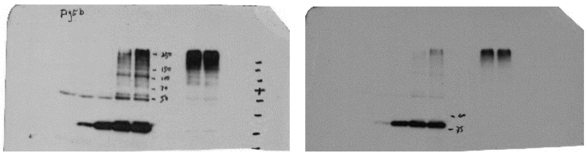

Figure 5c

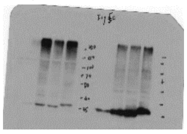

Figure 5d

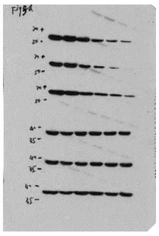

Figure 7b

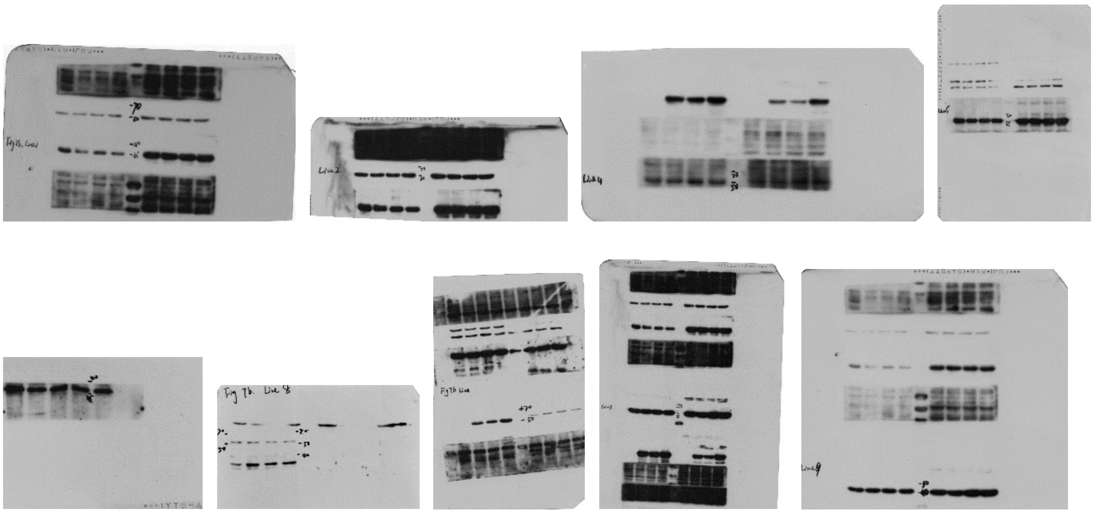

Figure 7g

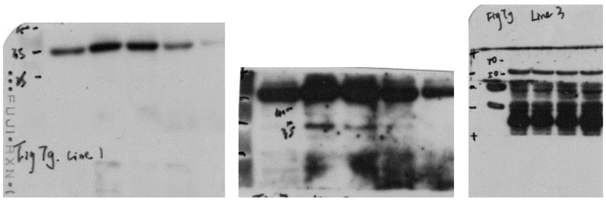

Supplementary Figure 2a

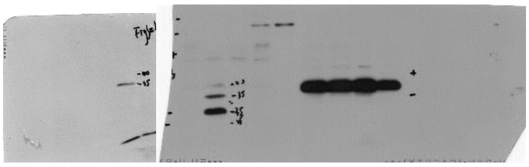

Supplementary Figure 2b

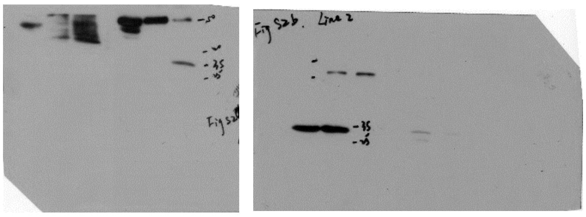

Supplementary Figure 2c

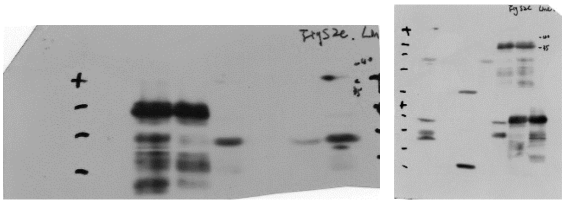

Supplementary Figure 2d

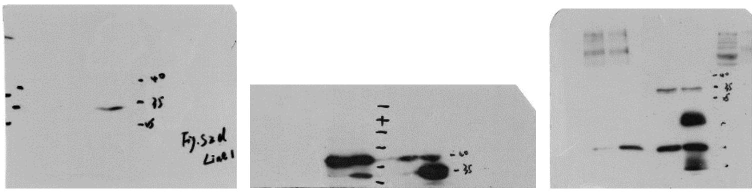

Supplementary Figure 3a

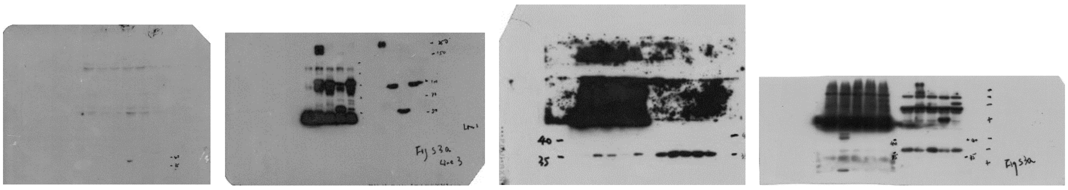

Supplementary Figure 3b

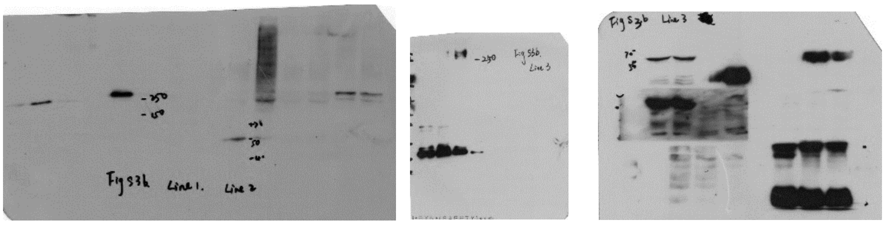

Supplementary Figure 3c

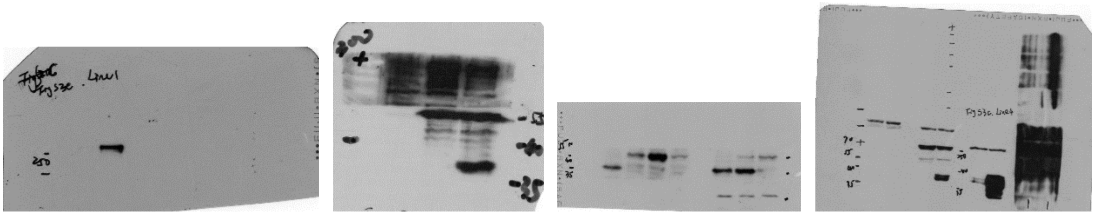

Supplementary Figure 3d

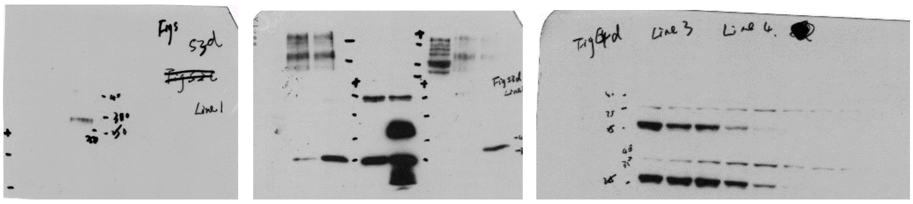

Supplementary Figure 3e

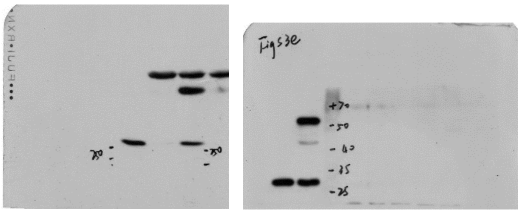

Supplementary Figure 5a

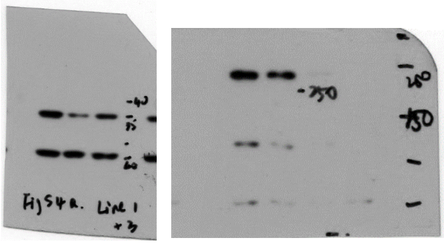

Supplementary Figure 5b

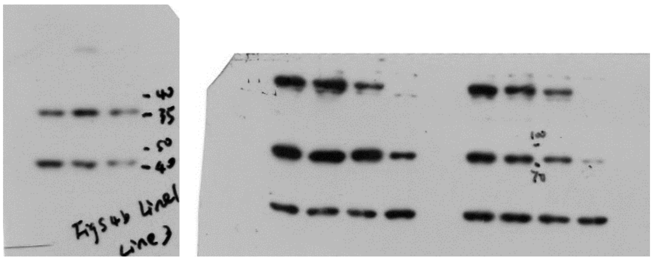

Supplementary Figure 5c

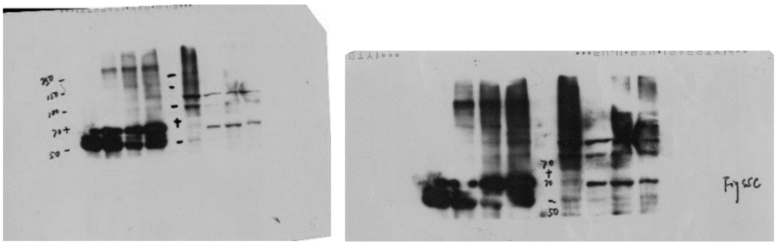

Supplementary Figure 6b

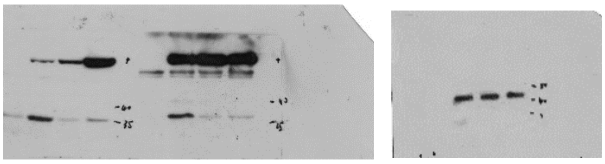

Supplement: Supplementary file 1 — Supplementary Information [file 41698_2021_158_MOESM1_ESM.pdf]
